# Lanthanide MOF-based luminescent sensor arrays for the detection of castration-resistant prostate cancer curing drugs and biomarkers†

**DOI:** 10.1039/d3sc06899d

**Published:** 2024-03-26

**Authors:** Xinrui Wang, Karuppasamy Gopalsamy, Gilles Clavier, Guillaume Maurin, Bin Ding, Antoine Tissot, Christian Serre

**Affiliations:** a Institut des Matériaux Poreux de Paris, Ecole Normale Supérieure, ESPCI Paris, CNRS, PSL University 75005 Paris France antoine.tissot@ens.psl.eu christian.serre@ens.psl.eu; b ICGM, Univ. Montpellier, CNRS, ENSCM 34095 Montpellier France; c Université Paris-Saclay, ENS Paris-Saclay, CNRS, PPSM 91190 Gif-sur-Yvette France; d Tianjin Key Laboratory of Structure and Performance for Functional Molecule, College of Chemistry, Tianjin Normal University 393 Binshui West Road Tianjin 300387 PR China hxxydb@tjnu.edu.cn

## Abstract

In recent years, castration-resistant prostate cancer (CRPC) has profoundly impacted the lives of many men, and early diagnosis of medication and illness is crucial. Therefore, a highly efficient detection method for CRPC biomarkers and curing drugs is required. However, the complex and diverse structures of CRPC drugs pose significant challenges for their detection and differentiation. Lanthanide metal–organic frameworks (Ln-MOFs) show great potential for sensing applications due to their intense and characteristic luminescence. In this work, a series of new bimetallic Ln-MOFs (Eu_*x*_Tb_1−*x*_-MOF) based luminescent sensor arrays have been developed to identify CRPC drugs, including in mixtures, *via* principal component analysis (PCA) and hierarchical cluster analysis (HCA) methods. These Ln-MOFs are built with a highly conjugated H_2_L linker (H_2_L = 5-(4-(triazole-1-yl)phenyl)isophthalic acid) and exhibit robust strong luminescence emissions (mainly located at 543 and 614 nm) and high energy transfer efficiencies. More specifically, Eu_0.096_Tb_0.904_-MOF (MOF 3) has demonstrated good sensing performances for CRPC curing drugs in real human serum samples. Furthermore, the curing drug hydroxyflutamide has been combined with MOF 3, to construct a robust composite sensing platform MOF 3@hydroxyflutamide for highly efficient detection of CRPC biomarkers such as the androgen receptor (AR) and prostate-specific antigen (PSA). Finally, luminescence lifetime measurements, zeta potential measurements, and density functional theory (DFT) calculations were performed to gain insights into the sensing mechanism.

## Abstract 2

In this work, we synthesized a novel luminescent array based on Eu/Tb-MOF, which was applied in the detection of drugs and biomarkers of castration-resistant prostate cancers with remarkable sensitivity and selectivity in human serum solution.

## Graphical abstract

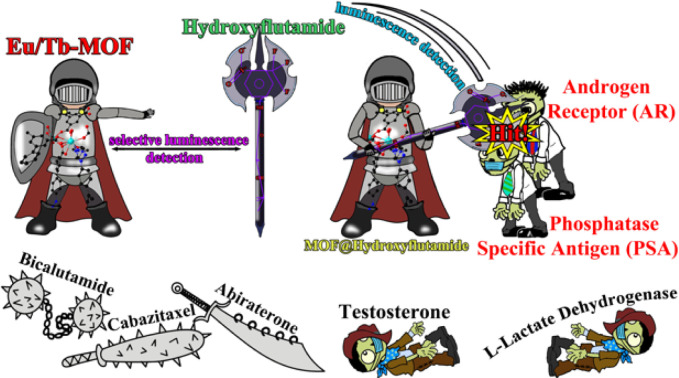

## Introduction

Prostate cancer (PC),^1^ currently the second most frequent cancer diagnosed in men and the fifth leading cause of death worldwide, affects more than 1.2 million patients each year.^2^ For PC patients, approximately 20% of men present advanced or metastatic disease.^3^ PC may be asymptomatic at the early stage and often has an indolent course that may require active surveillance.^4^ To enable early diagnosis, it is crucial to effectively distinguish PC biomarkers, with a particular focus on prostate-specific antigen (PSA).^5^ The concentrations of PSA are increased in 25% to 92% of patients with PC.^6^ The most accurate methods for detecting the PSA concentration currently involve a combination of rectal examination and serum PSA measurements (the concentration ranges between 1.0 and 1.5 ng mL^−1^ for healthy patients).^7^ At present, PSA is commonly detected by enzyme-linked immunosorbent assay (ELISA), radioimmunoassay (RIA), chemiluminescence immunoassay (LICA) and electrochemical luminescence immunoassay (ECLIA) as well as using diagnostic kits. These detection methods present a low limit of detection in the 0.005–0.05 ng mL^−1^ range and the associated limit of qualification can reach 0.01 ng mL^−1^.^8^ However, it is still required to reduce the pain that the patients must endure during the examination as well as to develop convenient techniques that doctors can quickly use to save time in diagnosing patients. Thus, the design of a fast and simple method to detect PSA in serum remains a challenge.

In the context of PC treatment, androgen deprivation therapy (ADT) has been the most basic method of care for the initial management of advanced or metastatic PC,^9^ but progression to castration-resistant prostate cancer (CRPC)^10^ may occur within 2–3 years of initiation of ADT. ADT can be performed through surgical removal of bilateral orchidectomy (surgical removal of both testicles) or medication (such as flutamide, cabazitaxel, bicalutamide, and abiraterone) aimed at reducing male hormone levels to combat PC, thereby curbing the growth and spread of cancer cells.^11^ Although ADT treatment has been confirmed as the main way to slow down the progression of CRPC, the five-year survival rate with ADT is only 41%^12^ and it easily led to serious side effects in patients such as sexual dysfunction, fatigue and/or metabolism disorder.^13^ In the context of CRPC, the androgen receptor (AR) remains an important driver of disease progression. AR gene amplification was found in 30% to 50% of CRPC patients, resulting in the overexpression of AR.^14^ Therefore, the AR can serve as a biomarker for CRPC, and the timely detection of the AR is invaluable for physicians to diagnose CRPC and mitigate additional suffering for patients.

In the past few years, the development of luminescent sensors^15^ has drawn extensive attention because of the great demand for analyzing complex analytes or unknown samples in the fields of environmental monitoring, disease diagnosis, food safety control, *etc.*^16^ Lanthanide metal–organic frameworks (Ln-MOFs) are 3D crystalline solids built from the assembly of organic linkers and Ln^3+^ cations that are attractive luminescent materials for sensing due to their remarkable luminescence properties such as large Stokes shift, high quantum yield and long lifetime.^17–19^ For example, Zeng *et al.*^20^ reported a single bimetallic Ln-MOF called Tb/Eu(BTB) (BTB = 1,3,5-benzenetribenzoic acid) as a powerful, versatile probe for fast and facile decoding of homologues, isomers, enantiomers, and even deuterated isotopomers with an energy transfer efficiency between Tb^3+^–Eu^3+^ of 69%. However, single emission-based Ln-MOFs suffer from drawbacks such as unstable luminescence emission signals and possible perturbations from the external environment.^21^ Consequently, achieving cross-reactive responses is crucial for discriminative sensors that aim to enable high-throughput detection or analysis of complex samples.^22^

Luminescent sensors, whether biological (*e.g.*, antibodies or enzymes) or chemical (*e.g.*, gas sensors), are designed to detect specific target analytes or molecules. However, due to similarities in structure or properties, they can sometimes respond to substances other than the primary target. This undesired response is known as cross-reactivity. However, realizing cross-reactivity with a single-sensor system is still highly challenging and single-system based discriminative sensors are less mature and efficient in comparison with multiple-element-based sensor arrays.^23,24^ To enhance selectivity, Ln-MOF based luminescent arrays^24^ have been designed, which may help estimate the patients' medication compliance.

Due to the adjustable porous structure of Ln-MOFs with diverse pore sizes/shapes, various chromophores, including organic dyes and carbon quantum dots, can be incorporated into Ln-MOF frameworks to fabricate luminescent arrays.^25^ Additionally, target molecules such as dye-labeled antibodies or DNA sequences can be attached on the surface of Ln-MOF to lead to anchored sensing platforms for analytes such as drugs or biomarkers. Additionally, luminescent arrays can consist of a series of Ln-MOFs with various emissions and unique fingerprint peaks to further enhance the selectivity. These arrays consist of multiple sensing units, each capable of interacting with various analytes and generating distinct luminescent signals in response. Through the analysis of combined response signals from multiple sensing units, the efficient detection of multiple analytes can be achieved. In comparison to conventional single luminescent sensors, luminescent arrays based on Ln-MOFs offer enhanced discrimination capabilities for multiple analytes with similar properties. For instance, Qian *et al.*^22^ successfully integrated Eu^3+^ and 3,3′-diethyloxacarbocyanine into MOF-253 to establish a luminescent array, demonstrating its effectiveness in identifying Ag^+^, Cu^2+^, Fe^3+^, Co^2+^, and Ni^2+^ within a mixture of metal ions.

**Scheme 1:**
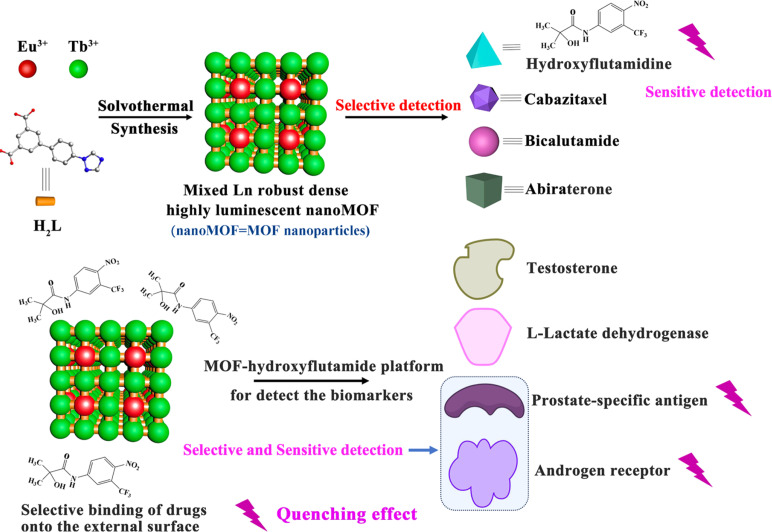
Illustration of the detection process of (top) CRPC curing drugs based on MOF 3; (bottom) CRPC biomarkers with MOF 3@hydroxyflutamide.

To get strong luminescence in Ln-MOFs, a dense phase with short Ln^3+^–Ln^3+^ distance is often required to facilitate efficient energy cascading.^26^ In addition, it is commonly admitted that a denser phase exhibits better chemical stability compared to a highly porous network, which is critical for practical applications involving contact with body fluids.^26,27^ In this work, we thus synthesized accordingly a series of new dense bimetallic Eu_*x*_Tb_1−*x*_-MOFs to construct luminescent arrays to discriminate and quantify CRPC effective drugs, which can also successfully estimate the components from blind CRPC curing drug samples (Scheme 1a). The detection and quantification of the specific biomarkers associated with CRPC are also crucial for effectively determining the progression of the disease. As hydroxyflutamide interacts with PSA and AR, we therefore construct the MOF 3@hydroxyflutamide sensing platform. It successfully achieved high-sensitivity detection of PC and CRPC biomarkers at extremely low concentrations, reaching detection limits (LOD) of 0.14 nM for the AR and 0.53 pM for PSA (Scheme 1b). A tentative detection mechanism is then proposed based on a series of advanced characterization techniques such as zeta potential measurements, and luminescence lifetime measurements as well as density functional theory (DFT) calculations.

## Results and discussion

To achieve a high energy transfer efficiency between the ions in the luminescent sensor, opting for a dense Ln-MOF structure (where Ln corresponds to lanthanide) is an appropriate choice because narrow pores favor proximity between the optical centers, which can promote energy cascading between Ln^3+^ ions. To serve this purpose, we chose the H_2_L ligand (H_2_L = 5-(4-(triazole-1-yl)phenyl)isophthalic acid) that possesses two carboxylic groups and a free nitrogen atom on a triazole ring, which can provide multiple coordinated sites for Ln^3+^ ions and hence favor the formation of a dense structure as Ln^3+^ cations are well known to exhibit a very high coordination number. In addition, our target MOFs are expected to present strong optical absorption in the UV region due to the strong aromaticity of the linker, paving the way for transferring UV excitation energy to Ln^3+^ to realize strong Ln^3+^ emission. Eu^3+^ and Tb^3+^ ions possess typical red and green emissions that can be observed in the visible region, which is a prerequisite for visual detection. Therefore, two new three-dimensional (3D) lanthanide MOFs were synthesized under solvothermal conditions using Eu^3+^ or Tb^3+^ salts and triazole dicarboxylic acid H_2_L (H_2_L = 5-(4-(triazole-1-yl)phenyl)isophthalic acid) (Fig. S1†). The crystal structures of (1) Eu-MOF [Eu_2_(L)_3_(DMF)_0.45_(H_2_O)_1.55_]_*n*_ and (2) Tb-MOF [Tb_2_(L)_3_(DMF)_0.25_(H_2_O)_1.75_]_*n*_ (DMF = *N*,*N*-dimethylformamide) were determined by single crystal X-ray diffraction analysis. Additionally, to obtain high energy transfer efficiency, a series of mixed-lanthanide frameworks with a controlled Eu/Tb ratio, namely, Eu_0.096_Tb_0.904_-MOF (3), Eu_0.051_Tb_0.949_-MOF (4), Eu_0.011_Tb_0.989_-MOF (5), Eu_0.415_Tb_0.585_-MOF (6) and Eu_0.516_Tb_0.484_-MOF (7) were prepared, and their relative metal contents were confirmed by inductively coupled plasma (ICP). The particle size of MOF 1–7 was finally adjusted to the nanoscale by tuning the reactant concentrations in order to prepare stable colloidal suspensions for a stable luminescent signal.

**Fig. 1:**
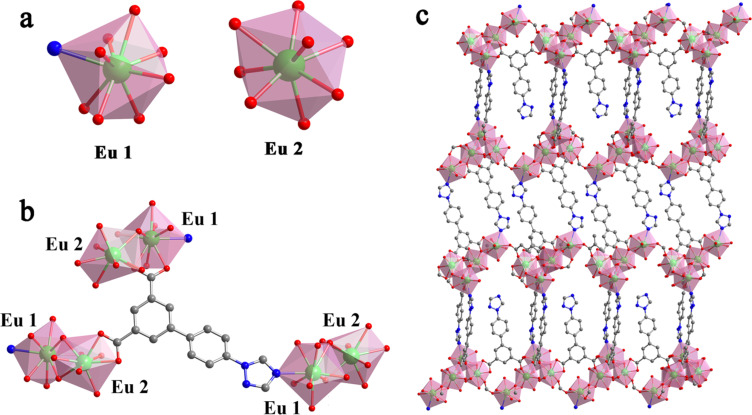
(a) Coordination environment of Eu ions in the structure of the Eu-MOF; (b) coordination mode of the H_2_L linker in the Eu-MOF; (c) 3D framework network of Eu-MOF.

Single crystal X-ray diffraction analysis showed that Eu-MOF and Tb-MOF are isostructural and crystallize in the monoclinic *C*2/*c* space group (no. 15). For the sake of simplicity, herein, we only describe the Eu-MOF (MOF 1) structure (Fig. 1, Table S1†).

The asymmetric unit of MOF 1 comprises two Eu^3+^ centers (Eu1 and Eu2). Eu1 is nine-coordinated to two oxygen atoms from coordinated water (namely O13) and DMF (namely O14) molecules respectively, six oxygen atoms (namely O1, O9, O10, O3, O4, and O5) belonging to the carboxylic group of the L^2−^ ligand and one nitrogen atom (N9) from the triazole group of the L^2−^ ligand. On the other hand, Eu2 is eight-coordinated to eight oxygen atoms from the L^2−^ ligand. Additionally, two Eu atoms (Eu1 and Eu1A or Eu2 and Eu2A) are interlinked *via* oxygen atoms from the L^2−^ ligand, leading to dinuclear Eu_2_O_2_ oxoclusters as secondary building units (SBUs). The distance between adjacent Eu^3+^ ions is 4.084 Å, which is good for building high electronic transfer efficiency. On the other hand, each fully deprotonated L^2−^ linker has three neighboring Eu_2_O_2_ SBUs with multidentate bridging mode (Fig. 1b), which ultimately constructs the oxocluster-based structure of MOF 1. The information on bond lengths and angles of MOF 1–2 is presented in the ESI (Tables S2–S5).† Eu–O bond lengths range from 2.309 Å to 2.608 Å, while the angles of O–Ln–O and N–Ln–O varied from 50.85° to 156.46°, in line with previous work.^13^ The Ln⋯Ln distances observed along the crystallographic *c* axis are 5.58 Å × 4.01 Å for MOF 1 and 5.55 Å × 4.01 Å for MOF 2. The shortest Ln^3+^–Ln^3+^ distance is 4.01 Å in the crystal structures, which suggests that an efficient energy transfer may occur between adjacent Ln centers upon light excitation. For example, the adjacent Ln^3+^ in Eu_*x*_Tb_1−*x*_-MOF are expected to promote energy cascading between Tb^3+^ and Eu^3+^, which might result in enhancing the emission of Eu^3+^ luminescence and may further improve the detection system sensitivity. Additionally, coordinated water molecules in MOF 1 and 2 can be involved in intermolecular C–H⋯O hydrogen bonds with carbon and oxygen atoms of L^2−^ (Tables S6 and S7†).

**Fig. 2:**
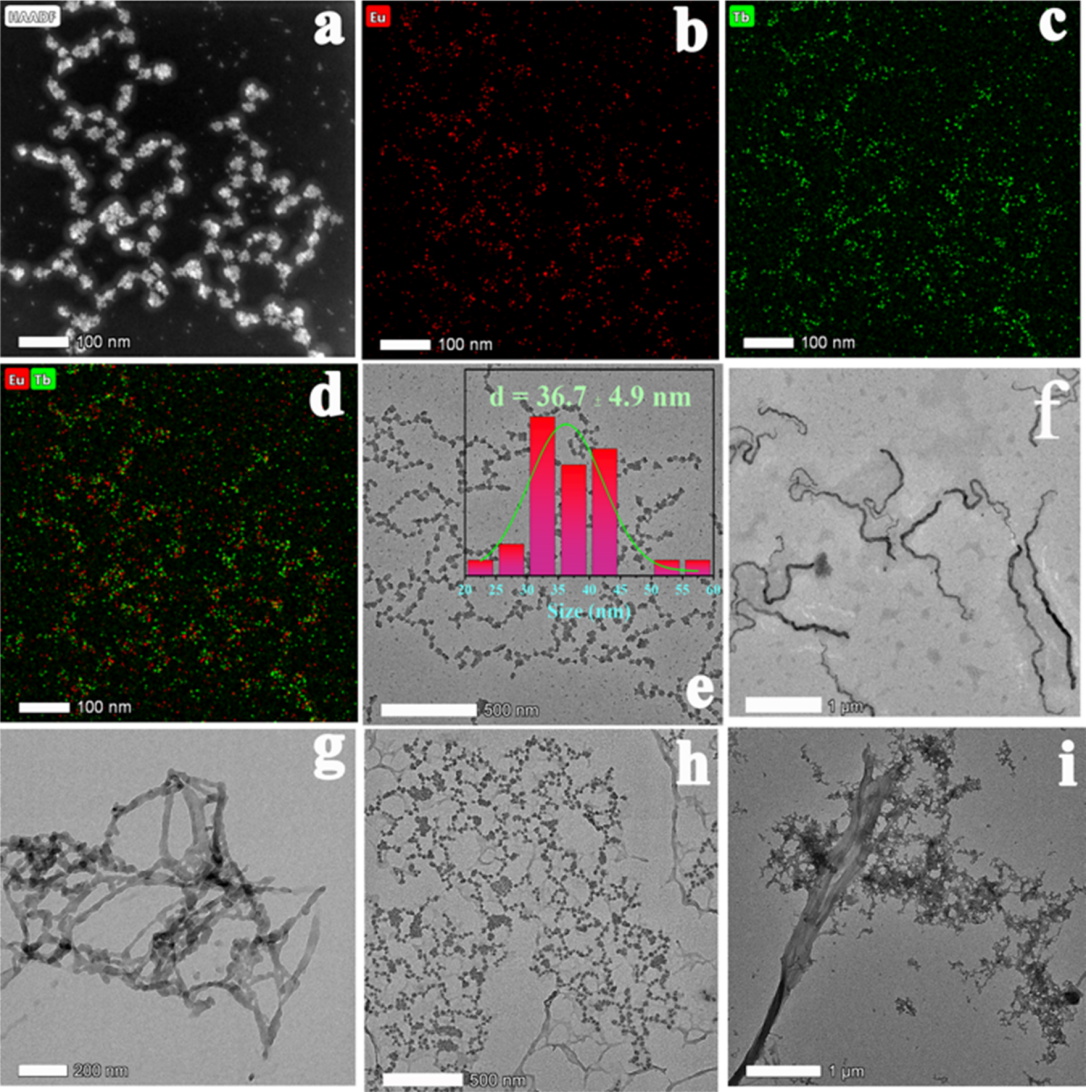
(a) STEM-HAADF image of MOF 3 nanocrystals; (b)–(d) EDS mapping images in STEM mode of MOF 3 nanocrystals; (e) TEM image of MOF 3 along with the corresponding particle size distribution; (f) TEM image of the AR; (g) TEM image of PSA; (h) TEM image of a mixture of AR, MOF 3 and hydroxyflutamide; (i) TEM image of a mixture of PSA and MOF 3@hydroxyflutamide.

Powder X-ray diffraction (PXRD) patterns of MOFs 1–7 (Fig. S2d†) confirm the phase purities of MOFs 1–7 in comparison with the theoretical ones. Thermogravimetric analysis (TGA) and variable temperature PXRD indicated that MOFs 1–3 are stable under an air atmosphere until 425 °C (Fig. S2a and b†). UV-vis absorption spectra showed that MOFs 1–3 possess a main adsorption peak located at 270 nm originating from H_2_L. Scanning transmission electron microscopy (STEM) combined with high angle annular dark field (HAADF) probes and energy dispersive spectroscopy (EDS) mapping was performed and demonstrates that Eu^3+^ and Tb^3+^ are uniformly dispersed in the nanocrystals of MOF 3 (Fig. 2).

MOFs 1–7 exhibited strong and narrow photo-luminescence emission peaks, which is in line with the excellent emission properties of Ln-based luminescent probes.^29^ When excited at 250 nm, Eu-MOF (1) displayed two main and strong emission peaks located at 593 and 617 nm (Fig. S3†) attributed to ^5^D_0_ → ^7^F_1_ and ^5^D_0_ → ^7^F_2_ transitions of Eu^3+^, while Tb-MOF (2) possessed four emission peaks located at 492, 544, 583 and 618 nm (Fig. S3†), which should be attributed to ^5^D_4_ → ^7^F_*J*_ (*J* = 3–6) transitions of Tb^3+^. MOFs 3 and 4 exhibited the emissions of both Tb and Eu ions with two strong emissions at 543 and 617 nm respectively, similarly to previous studies.^34^ Therefore, MOFs 1–4 were selected to construct luminescent arrays to carry out the discrimination task of CRPC effective drugs. High absolute quantum yields for MOF 1–4 (21% for Eu-MOF (1), 14% for Tb-MOF (2), 42% for MOF 3 and 38% for 4) could be ascribed to the strongly conjugated backbone of H_2_L constructed with triazole and bi-phenyl aromatic moieties and short Ln–Ln distances. Luminescence lifetime measurements were performed at 617 and 543 nm, corresponding to Eu^3+^ and Tb^3+^ main emission peaks respectively, in single metal and all bimetallic Ln-MOFs (MOFs 3–7) (Fig. S4†). In the latter, ^5^D_4_ (Tb^3+^) lifetime in MOFs 3–7 was found to be shorter than that in pure Tb-MOF (2) (153 μs) while ^5^D_0_ (Eu^3+^) lifetime in MOFs 3–7 was longer than that in pure Eu-MOF (1) (98 μs), suggesting the presence of a Tb^3+^-to-Eu^3+^ excitation energy transfer process. It is noteworthy that MOF 3 exhibited a very long lifetime of 544 μs, in line with high energy transfer efficiency between Tb^3+^ and Eu^3+^. This long lifetime will improve the sensitivity in the detection process by enhancing the emission of Eu^3+^ and Tb^3+^ in MOF 3, making easier the observation of the emission variation of MOF 3 after adding trace amounts of analytes.^28^ Therefore, MOF 3 was selected for ratiometric luminescence sensing to detect CRPC biomarkers and MOF 1–4 were selected as luminescent arrays to recognize CRPC drugs.

**Fig. 3:**
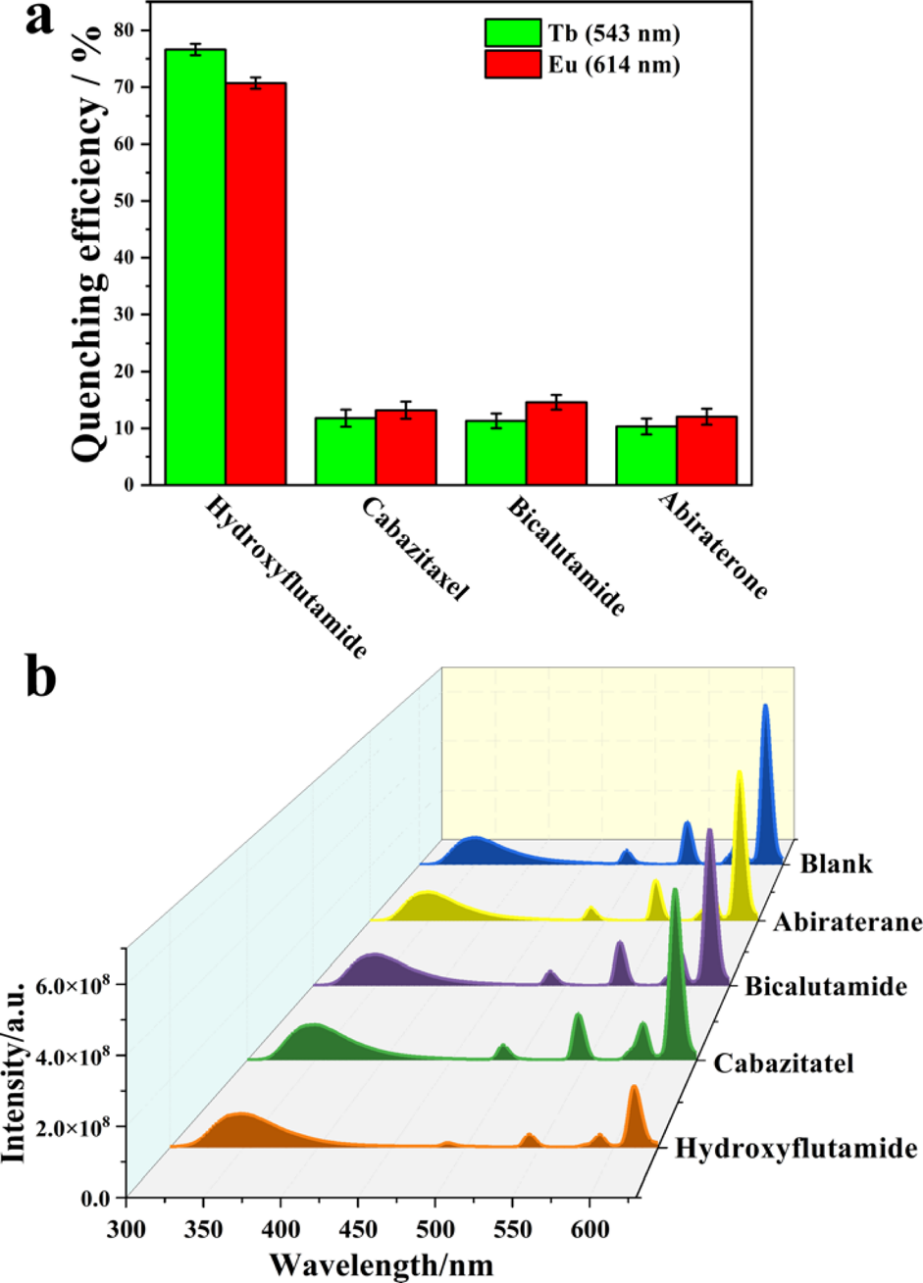
(a) Quenching efficiency of CRPC effective drugs (abiraterone, bicalutamide, cabazitaxel, and hydroxyflutamide) towards MOF 3; (b) luminescence spectra of 3 before (blank) and after adding abiraterone, bicalutamide, cabazitaxel, and hydroxyflutamide, (excitation position: 250 nm) in the DMSO solution, test interval: 5 min.

**Fig. 4:**
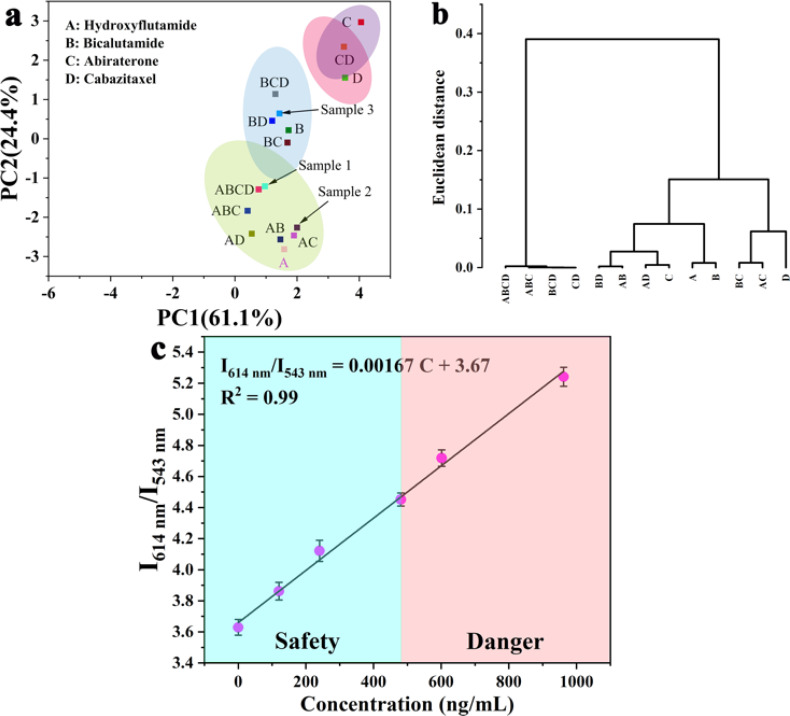
(a) PCA and (b) hierarchical cluster analysis (HCA) plot for the discrimination of the 13 analytes based on the relative luminescence quenching effect of the Ln-MOF sensor array. (c) A linear relationship between the concentration of hydroxyflutamide and the luminescence intensity ratio of MOF 3.

The luminescent sensing strategy^29–32^ was here expected to provide sensitivity to recognize hydroxyflutamide based on ratiometric luminescent sensor MOF 3. In this context, only hydroxyflutamide exhibited the most pronounced quenching effect on the emission of Eu^3+^ (at 614 nm) and Tb^3+^ (at 543 nm) in MOF 3 compared to other drug solutions (Fig. 3). The observed intensity changes at maximum emission were fitted using Stern–Volmer equation *I*_1_/*I*_2_ = *K*_SV_[C] + *M*, where [C] represents the analyte concentration and *K*_SV_ is the quenching constant (Fig. S5,†*I*_614 nm_/*I*_543 nm_ = 4.98*C* + 3.69), leading to a *K*_SV_ of 4.98 × 10^12^ [M]^−1^. Based on these results, we could also calculate the limit of detection (LOD) of Ln-MOFs toward hydroxyflutamide (8.37 fM), according to the relation: LOD = 3*S*_0_/*S* (*S*_0_ is the standard deviation of luminescence intensity of blank for ten times and *S* is the slope of the calibration curve, which corresponds to *K*_SV_). This compound was found to be much more sensitive than other existing sensors (Table S9†), while the recovery rate was 95.3–105.6% in human serum solution, demonstrating the high sensitivity of the compound (Table S10†). It is well recognized that excessive use of hydroxyflutamide can lead to serious side effects such as male breast development and/or tenderness, sometimes accompanied by galactorrhea, abnormal liver function, and hepatitis. To avoid this dangerous situation, one of the best methods is to monitor the critical concentration in human serum, which is also represented by the maximum blood concentration (*C*_max_). After adding different concentrations of hydroxyflutamide, we could obtain a linear relationship between the concentration of hydroxyflutamide in human serum and the luminescence ratio (*I*_614 nm_/*I*_543 nm_) of MOF 3 (*I*_614 nm_/*I*_543 nm_ = 0.00167*C* + 3.67, *C* represents the concentration of hydroxyflutamide) in the concentration range of *C*_max_ (480.9 ng mL^−1^) (Fig. 4). MOF 3 can therefore be considered a ratiometric luminescent sensor, suitable for the practical detection of hydroxyflutamide.

Flutamide, cabazitaxel, bicalutamide, abiraterone and different mixtures of these drugs were chosen as models to explore the discrimination ability of the MOF 1–4 based sensor array (Fig. S3†). Principal component analysis (PCA) was employed to analyze the responses of these MOF sensors in luminescence titration experiments. PCA is a popular technique for analyzing large datasets containing a high number of dimensions/features per observation, increasing the interpretability of data while preserving the maximum amount of information, and enabling the visualization of multidimensional data.^29,30^ An effective multidimensional sensor is expected to accurately monitor and identify targeted analytes by leveraging multiple transduction channels.^30^

Herein, we probed the variation of the fluorescence intensity ratio after adding different drugs to MOF 1–4 and UV-vis absorption spectra of CRPC drugs were subjected to canonical factors. Then, the response patterns of each drug were distinguished by PCA because of its excellent description of the sample. Noteworthily, two canonical factors (PC 1 and PC 2) were used to obtain a two-dimensional plot as shown in Fig. 4a, which demonstrated that each combination of drugs could be well-identified and discriminated against from each other. Additionally, to further improve the identification ability of the luminescent array for CRPC drugs, 13 different types of CRPC drugs (0.02 mM) were classified by hierarchical cluster analysis (HCA) into different clustering groups, which were shown to be consistent with the PCA result (Fig. 4b). All these above statistical analysis results proved that the sensor arrays could identify a variety of CRPC drugs with a satisfactory selectivity. To further evaluate the practicality of this MOF based sensor array, three unknown samples were analyzed using this sensing platform. Remarkably, their luminescent response analyzed by PCA matched well the real samples composition, which demonstrated that our new sensor array is a promising candidate to analyze real samples.

Density functional theory (DFT) calculations were finally further performed to shed light on the preferential interactions between hydroxyflutamide and Eu-MOF (MOF 1).

**Fig. 5:**
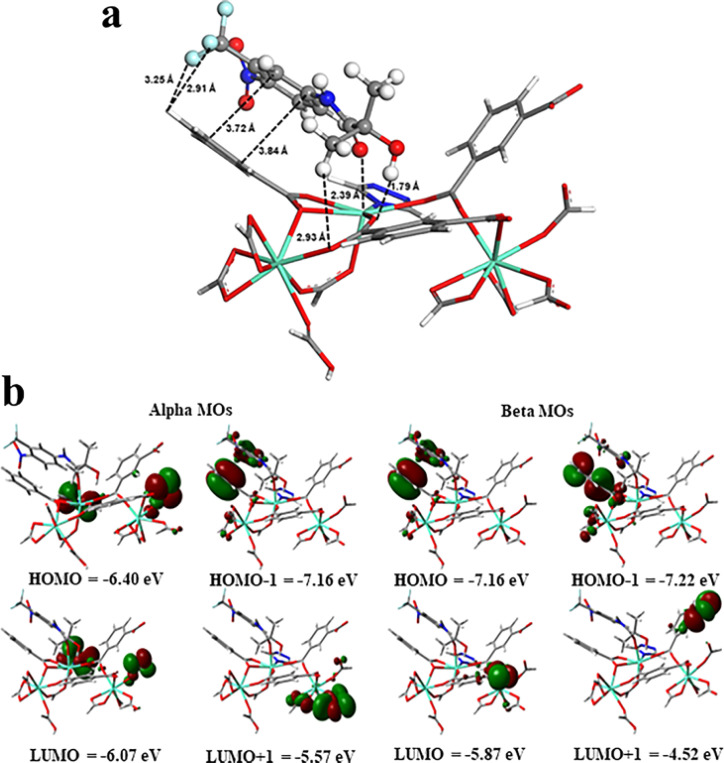
(a) Optimized DFT geometry of the hydroxyflutamide molecule (ball and stick) interacting with the Eu-MOF cluster (stick). The characteristic interacting distances are reported in Å. Color code for all atoms: hydrogen (white), carbon (light grey), nitrogen (blue), oxygen (red), fluorine (cyan), and europium (turquoise); (b) the alpha and beta frontier molecular orbitals (HOMO (HOMO − 1) and LUMO (LUMO + 1)) diagrams for the MOF 1@hydroxyflutamide (stick). In the MOs, red and green colors indicate the negative and positive charges, respectively.

The molecular dimensions of hydroxyflutamide largely exceed the pore size of MOF 1 (the pore limiting diameter is 6.38 Å and largest cavity diameter is 12.8 Å, where the size of hydroxyflutamide is 12.8 Å × 8.2 Å × 6.2 Å) and therefore, this molecule is expected to interact only with the external surface of the MOF nanoparticles. Therefore, a cluster model containing 3 Eu^3+^ open metal sites potentially present at the MOF surface and free to interact with hydroxyflutamide was then cleaved from the crystal structure and loaded with 1 hydroxyflutamide molecule. This cluster model mimics the local environment of Eu^3+^ open metal sites in the MOF crystal and exposes these open metal sites to plausible interactions with the guest molecule that are likely to proceed *via* the MOF surface. Multiple starting orientations of the hydroxyflutamide molecule within the MOF cluster were considered corresponding to two possible binding modes (i) a single adduct *via* their carbonyl function and (ii) chelating species implying both carbonyl and hydroxyl functions to ensure efficient sampling of the electronic potential energy surface. All the electronic structure calculations were performed using the Gaussian 09 suite of program,^33^ employing the B3LYP functional^34,35^ along with Grimme's D3-BJ correction.^36,37^ The 6-31G(d) basis set^38^ was employed for H, C, N, O and F atoms, while a 28-electron quasi relativistic effective core potentials and the segmented basis set MWB28 were used for Eu^3+^ metal ions.^39,40^ A total spin multiplicity (2*s* + 1) of 19 for the cluster was considered with high spin configuration for the three Eu^3+^ atoms present in the MOF cluster model. In all these calculations, atoms of the MOF cluster were maintained fixed in their original positions in order to maintain the geometry of the corresponding MOF while the hydroxyflutamide molecule was relaxed freely without any constraints. Fig. 5a reveals that hydroxyflutamide adopts a preferential orientation in such a way to favor multiple interactions with the MOF cluster including (i) a predominant interaction between its carbonyl group and the Eu^3+^ metal site associated with a relatively short O_(CO)_⋯Eu^3+^ distance of 2.39 Å, (ii) a side interaction between its –OH group and the carboxylate oxygen atom H_(OH)_⋯O_(COO)_ with an associated separating distance of 1.79 Å, (iii) π–π stacking (Van der Waals) between its phenyl ring and the organic linker as well as (iv) additional hydrogen-bonding between its functional groups and different atoms of the MOF cluster, CH_3_⋯O_(COO)_, CF_3_⋯H_(phenyl)_ and NO_2_⋯H_(phenyl)_, respectively. The resulting single adduct is likely due to the steric hindrance around the Eu^3+^-metal sites preventing the formation of a chelation geometry. The calculated interaction energy for the resulting MOF 1@hydroxyflutamide adduct is −86.6 kJ mol^−1^, in line with the good MOF 1@hydroxyflutamide affinity observed experimentally. Frontier molecular orbitals (MOs) were further analyzed to evaluate the stability of the MOF 1@hydroxyflutamide adduct. Fig. 5b illustrates the charge density localization and energy transport within the alpha and beta MOs of the MOF 1@hydroxyflutamide adduct. For both MOs, HOMOs (HOMO − 1) were associated with the following energies: −6.40 eV (−7.16 eV) and −7.16 eV (−7.22 eV) and exhibit a total charge density localized over the imidazole and phenyl rings.

To discuss the mechanism of the hydroxyflutamide detection process, we needed to consider several points: first, from the DFT calculation result, we excluded the possibility of charge or electron transfer from H_2_L in MOF 3 to hydroxyflutamide in the detection process. Additionally, the PXRD pattern of MOF 3 after adding different analytes did not change (Fig. S2d†), which means that MOF 3 was stable during the detection process, ruling out the collapse of its framework as the source of the quenching effect. Compared with other drugs, only hydroxyflutamide possesses “–NO_2_” and “–CF_3_” electron-withdrawing groups, that can be involved in the quenching mechanism through photoinduced electron transfer,^41,42^ which explains why MOF 3 can carry out selective detection of hydroxyflutamide in a mixture of other drugs. From the luminescence decays' experiments, the luminescence lifetime of MOF 3 at 543 nm decreased from 714 μs to 664 μs and decreased from 428 μs to 353 μs at 614 nm after adding a similar concentration of hydroxyflutamide, while the lifetime of MOF 1 located at 614 nm decreased from 131 μs to 129 μs and the lifetime of MOF 2 located at 543 nm decreased from 225 μs to 213 μs (Fig. S6, Table S11†). This indicated that hydroxyflutamide gives strong interference during the Tb^3+^ to Eu^3+^ energy transfer process in MOF 3, which leads to a strong quenching effect. Therefore, the main factor in the detection mechanism is that hydroxyflutamide can influence the energy transfer process between the Ln^3+^ ions.

**Fig. 6:**
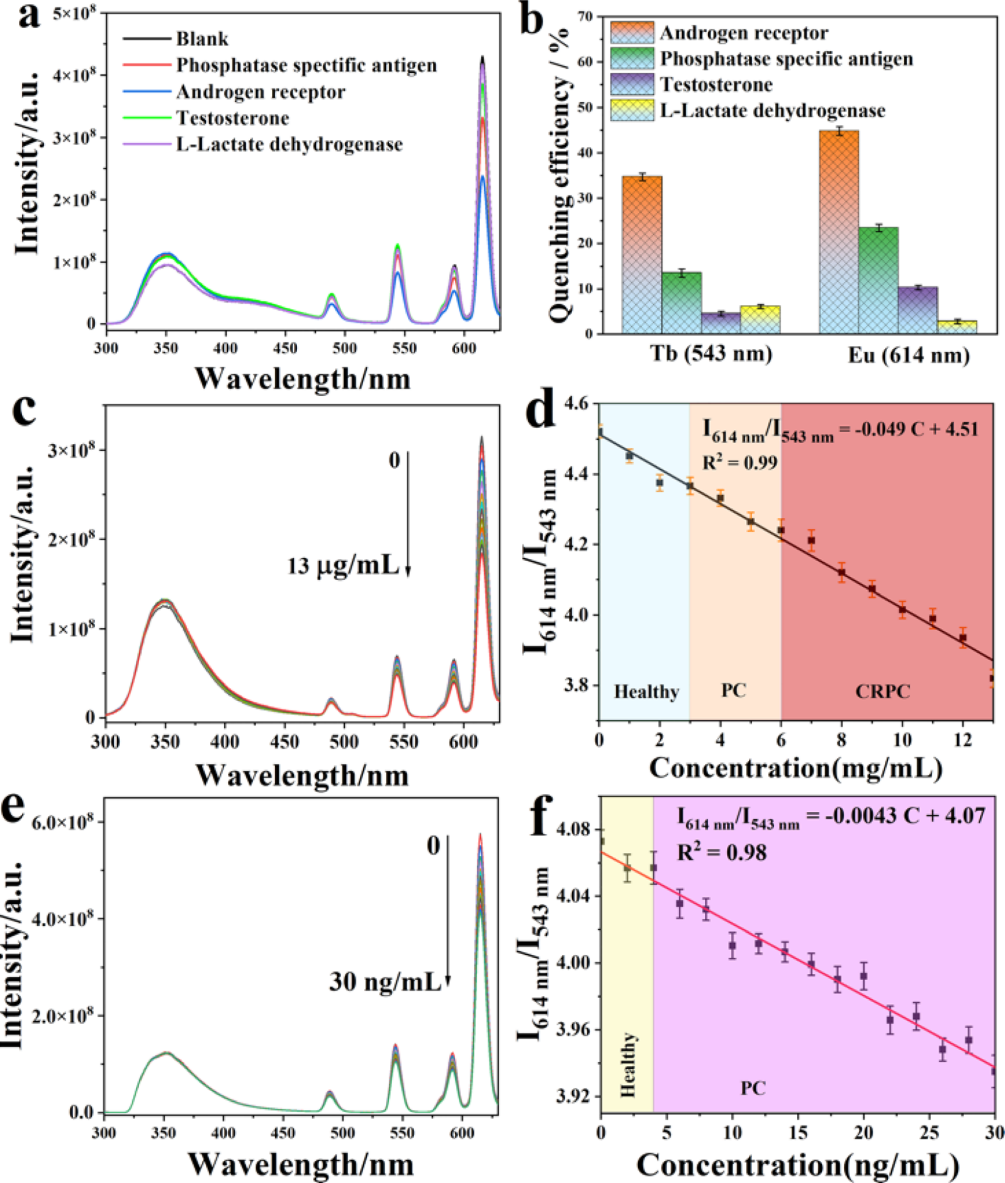
(a) Luminescence spectra of MOF 3@hydroxyflutamide (0.15 pM) before (blank) and after adding phosphatase specific antigen (PSA), androgen receptor (AR), testosterone and l-lactate dehydrogenase, (excitation position: 250 nm) in human serum solution after 5 minutes of incubation; (b) quenching efficiency of CRPC biomarkers (PSA, AR, testosterone, and l-LDH) towards MOF 3@hydroxyflutamide. (c) Luminescence spectra of MOF 3@hydroxyflutamide after adding the AR at different concentrations (excitation: 250 nm); (d) linear relationship between concentration of the AR and relative luminescence intensity ratio of MOF 3@hydroxyflutamide; (e) luminescence spectra of MOF 3@hydroxyflutamide after adding PSA at different concentrations (excitation: 250 nm); (f) linear relationship between the concentration of PSA and relative luminescence intensity ratio of MOF 3@hydroxyflutamide.

To discuss selectivity studies of CRPC biomarkers, specific recognition of the target over other species is also necessary for real conditions, *i.e.* in a complex biological matrix. Interestingly, our luminescent MOF 3@hydroxyflutamide platform displayed a good selectivity toward the AR and PSA. As shown in Fig. 6, the luminescence emission of MOF 3@hydroxyflutamide decreases indeed in the presence of the AR and PSA, while other CRPC biomarkers such as testosterone and l-lactate dehydrogenase (l-LDH) did not induce noticeable changes in luminescence. Additionally, the luminescence of MOF 3 without hydroxyflutamide could not be quenched by the AR or PSA (Fig. S7 and S8†). To evaluate the potential of MOF 3@hydroxyflutamide for detecting PSA and the AR under real conditions, additional experiments were carried out in 10% human serum solution (water as solvent). Remarkably, linear relationships between the luminescence intensity ratio of Eu^3+^ and Tb^3+^ in MOF 3@hydroxyflutamide (represented by *I*_614 nm_/*I*_543 nm_) at different concentrations of biomarkers were obtained. For the detection of PSA, a good linear correlation was observed (*I*_614 nm_/*I*_543 nm_ = −0.049*C* + 4.07) in the 0 to 30 ng mL^−1^ range with a coefficient of determination of *R*^2^ = 0.98 (Fig. 6) in a mixed biomarker solution (containing testosterone and l-LDH and the AR; the concentration of them is located in a normal range). This relationship could be further applied to monitor the concentration of PSA in body fluids to determine if a patient is at risk of PC. Therefore, MOF 3@hydroxyflutamide can realize specific detection for the AR and PSA among other CRPC biomarkers.

To test the sensitivity of MOF 3@hydroxyflutamide towards PSA or AR, we first investigated the sensitivity of MOF 3@hydroxyflutamide towards PSA (Fig. 6). The quenching constant *K*_SV_ of PSA was evaluated at 7.9 × 10^10^ [M^−1^] (Fig. S9†), and LOD for PSA is 15.9 pg mL^−1^, which is far more sensitive than that in previous studies (Table S13†). For the AR detection, we could get a linear relationship between the concentration of the AR and intensity ratio of MOF 3@hydroxyflutamide; the equation was *I*_614 nm_/*I*_543 nm_ = −0.0043*C* + 4.07, and the detection range is 0–30 ng mL^−1^. When one investigates the concentration of AR, we can infer whether the patient has PC or CRPC. The quenching constant *K*_SV_ of the AR was evaluated at 2.9 × 10^8^ [M^−1^](Fig. S10†), and the limit of detection (LOD) for the AR is 0.14 nM. Therefore, MOF 3@hydroxyflutamide can realize highly sensitive detection towards the AR and PSA.

Then, to understand the mechanism of the CRPC biomarker sensing, we checked if our MOF was stable under the testing conditions. According to TEM images, the biomarkers should interact with the surface of MOF 3 nanoparticles (Fig. 2), which indicates that the biomarkers' detection process happens on the surface of MOF 3. We then probed the charge of the MOF particles to explore the possible electrostatic interactions between the MOF and the analytes. After adding hydroxyflutamide and PSA, zeta potential of MOF 3 turned from −6.1 mV to −0.87 mV (Fig. S11†), while after adding hydroxyflutamide and AR, zeta potential of MOF 3 increased from −6.1 mV to 4.9 mV, which implies that electrostatic interactions exist between MOF 3, hydroxyflutamide and the AR or PSA, which further lead to a quenching effect in the biomarkers' detection process. A similar effect was already observed by Wang *et al.*^18^ who combined a Y-based MOF with a DNA sequence to form a composite able to detect COVID-19 biomarkers through a quenching effect due to electrostatic interactions that leads to a decrease in energy transfer efficiency. In Raman spectra (Fig. S11a†), during the PSA detection, the peak located at 1667 cm^−1^ (attributed to the β-strand in PSA)^44,45^ has red shifted to 1678 cm^−1^, and the peak intensity has been gradually increased^45^ after adding MOF 3@hydroxyflutamide. This phenomenon is similar to AR detection (Fig. S11b†): the peak located at 1631 cm^−1^ (attributed to α-helical in AR) has red shifted to 1654 cm^−1^, and peak intensity has gradually increased after adding MOF 3@hydroxyflutamide. This demonstrates that hydrogen bonding interaction between MOF 3, hydroxyflutamide, PSA, or AR has been strengthened;^44^ consequently, this further leads to a quenching effect towards MOF 3.^44,45^ The –OH” group in hydroxyflutamide may be considered to interact with naked l-serine in PSA or bare lysine and glutamic acid through hydrogen bonding interaction.^43,44^ Additionally the ligand H_2_L in MOF 3 also further provides strong π⋯π interaction with hydroxyflutamide and biomarkers. Thus MOF 3@hydroxyflutamide can be used as an efficient detection system for PSA or AR.

**Scheme 2:**
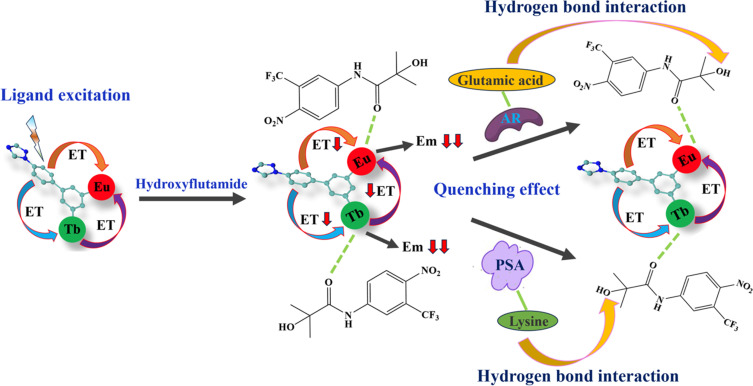
Sensing mechanism of MOF 3@hydroxyflutamide towards the AR and PSA (ET = energy transfer and Em = emission).

We then analyzed the luminescence decay (Fig. S13–S15†): after adding PSA, the lifetime of MOF 1@hydroxyflutamide (emission at 614 nm) decreased from 234 μs to 189 μs (Table S14†) while the one of MOF 2@hydroxyflutamide (emission at 543 nm) decreased from 208 μs to 194 μs. The lifetime of MOF 3@hydroxyflutamide at 614 nm decreased from 234 μs to 0.14 μs and MOF 3@hydroxyflutamide located at 543 nm increased from 125 μs to 428 μs. These results are clear indications that PSA interrupts the energy transfer between Tb^3+^ and Eu^3+^ in MOF 3@hydroxyflutamide. Similar results were obtained for AR detection (more details in Section 5, ESI†). We also excluded that PSA or the AR might influence the energy transfer from the H_2_L ligand in MOF 3 to Ln^3+^ after adding analytes, as the luminescence lifetime of MOF 3@hydroxyflutamide at 350 nm nearly did not change (Table S16†). Therefore, hydrogen bonding interactions, electrostatic interactions, and energy transfer interruption between Tb^3+^ and Eu^3+^ in MOF 3 are undoubtedly at the origin of the selective sensing mechanism during CRPC biomarker detection (Scheme 2).

## Conclusion and perspective

In this work, we report for the first time a highly sensitive and selective ratiometric luminescent sensor towards the CRPC drug hydroxyflutamide based on a new water stable dense bimetallic Eu/Tb-MOF constructed from a highly conjugated ligand. This nonporous MOF, stable under body fluid conditions, was utilized to construct a series of luminescent arrays able to efficiently discriminate various CRPC drugs. Finally, when combined with hydroxyflutamide, our resulting MOF@hydroxyflutamide could detect CRPC biomarkers PSA and AR with high sensitivity under real conditions (concentration, mixtures, and body fluids). The sensing mechanisms of CRPC drug and biomarker detection have been deciphered using hydrogen bonding interactions, electrostatic interactions, and energy transfer interruption between Tb^3+^ and Eu^3+^ in the MOF. These results pave the way for the development of novel detection devices for drugs *via* luminescent bimetallic MOFs and we hope more scientists can benefit from designing new luminescent arrays to realize drug reorganization and will help MDs make CRPC diagnostic to decrease the death rate. Moreover, Ln-MOFs could be combined with different antibodies or target DNA molecules in order to form multifunctional luminescent arrays to detect specific biomarkers.

## Data availability

The data supporting this article have been uploaded as part of the ESI.†

## Author contributions

The original idea was conceived and experimental data analysis was performed by Xinrui Wang under supervision of Antoine Tissot, Christian Serre, Bin Ding and Gilles Clavier. Structure characterization, quantum yield measurements, TEM and Raman spectroscopy were performed by Bin Ding. Luminescence decay experiments were performed by Gilles Clavier. DFT calculations were carried out by Karuppasamy Gopalsamy under supervision of Guillaume Maurin. The original manuscript was first drafted by Xinrui Wang and revised by the main authors. All authors have participated in modification of the manuscript and given approval to the final manuscript.

## Conflicts of interest

There are no conflicts to declare.

## Supplementary Material

SC-015-D3SC06899D-s001

SC-015-D3SC06899D-s002
