# Supplementary material for: Lanthanide MOF-based luminescent sensor arrays for the detection of castration-resistant prostate cancer curing drugs and biomarkers
Source: Chem Sci. 2024 Mar 26;15(17):6488–99. doi: 10.1039/d3sc06899d (PMC11062119; doi:10.1039/d3sc06899d)
Supplement: SC-015-D3SC06899D-s001 [file SC-015-D3SC06899D-s001.pdf]

Electronic Supplementary Material (ESI) for Chemical Science.
This journal is © The Royal Society of Chemistry 2024

Supporting information

Lanthanide MOF Based Luminescent Sensor Arrays for the Detection of Castration-Resistant Prostate Cancer Curing Drugs and Biomarkers

Xinrui Wang[a], Karuppasamy Gopalsamy [b], Gilles Clavier[c], Guillaume Maurin[b], Bin Ding*[d], Antoine Tissot*[a] and Christian Serre*[a]

[a] Institut des Matériaux Poreux de Paris, Ecole Normale Supérieure, ESPCI Paris, CNRS, PSL University, 75005 Paris, France.
[b] ICGM, Univ. Montpellier, CNRS, ENSCM, 34095 Montpellier, France.
[c] Université Paris-Saclay, ENS Paris-Saclay, CNRS, PPSM, 91190 Gif-sur-Yvette, France.
[d] Tianjin Key Laboratory of Structure and Performance for Functional Molecule, College of Chemistry, Tianjin Normal University, 393 Binshui West Road, Tianjin 300387, PR China

1. General methods

The H_2L Ligand was purchased from JiNan Henghua (Jinan, China). DNA sequences were purchased from AZENTA company. All the other reagents were commercially purchased and utilized without further purification. C, H, and N microanalyses were carried out with a Perkin-Elmer 240 elemental analyzer. Powder X-Ray Diffraction (PXRD) was characterized by a high-throughput Bruker D8 Advance diffractometer working on transmission mode and equipped with a focusing Göbel mirror producing CuKα radiation (λ = 1.5418 Å) and a LynxEye detector. FT-IR spectra (4000- 500 cm^{-1}) were recorded by using a Nicolet iS5 FTIR ThermoFisher spectrometer. Ultrasonic preparation was carried by a Branson Ultrasonic bath (sonifier sound enclosure Instrument, USA). Agilgent technologies (USA) cary 300 spectrophotometer was utilized to record ultraviolet-visible (UV-vis) adsorption spectra. The photo-luminescent spectra were performed using a Fluorolog-3, Horiba Jobin Yvon (USA). TEM, STEM-HAADF, and EDS images were recorded with Thermo Fisher Scientific Talos F200X (USA), spot size of the beam used for TEM-EDS imaging is 6.

Lifetime experiments were done using 10 mm path length quartz cuvettes: The ns fluorescence decay curves were obtained by the time-correlated single-photon counting (TCSPC) method. The setup is composed with a titanium sapphire Ti:Sa oscillator (Spectra Physics, Maï Taï HP) emitting pulses of 100 fs duration at 690nm, 80MHz frequency. The laser pulses then pass through a pulse picker which implements acousto-optic modulator to pick up specific pulse to reduce the repetition rate at 4MHz (GWU Lasertechnik, UHG-23-PSK). Then the beam, after adjusting the excitation power and the polarization with respectively an intensity attenuator filter wheel and a Fresnel rotator, passes through the sample solution. Fluorescence photons were detected at 90° through a long pass filter (Schott, RG9), a monochromator (CVI Laser Corporation, Digikröm CM110) and a polarizer by means of a micro channel plate photomultiplier (Hamamatsu, MCP-PMT R3809U-50), connected to a TCSPC module (Becker & Hickl, SPC-630). Time-correlated fluorescence decay data is finally processed and analysed with the help of a software which implements the non-linear square method (Globals, Laboratory for Fluorescence Dynamics at the University of Illinois at Urbana-Champaign). The Absolute quantum yield test of MOFs 1-4 were measured on a SPEX Fluoromax-3 (Horiba Jobin-Yvon) equipped with an integrating sphere of 10 cm diameter and a Horiba Jobin-Yvon acquisition and analysis procedure. Quantum yields were calculated using equation (1), where the subscript with "0" and "x" stand for the corresponding parameter for the standard and sample and Φ_F, A, S and n represents the quantum yield, absorbance, integrated photoluminescence intensity and refractive index, respectively. For this measurement, the excitation position is 260 nm and the emission range is from 535 nm to 620nm. Zeta potential measurements were performed on Nano-ZS, Worcestershire, (Malvern, UK). MilliQ water was obtained from Millipore system. TGA data were collected on Mettler Toledo TGA/DSC 2, STAR System apparatus with a heating rate of 5 °C/min under the oxygen flow. ICP analysis were carried by Agilent 7700 Series ICP-MS, USA.

Equation 1:

$$\Phi_F(x) = \Phi_F(0)\,\frac{1-10^{-A_0}}{1-10^{-A_x}}\,\frac{S_x}{S_0}\left(\frac{n_x}{n_0}\right)^2$$

2. X- ray Crystallography.

Single crystal X-Ray diffraction experiments where performed on MOF 1 and MOF 2 on a Bruker

SMART 1000 CCD diffractometer utilizing graphite-monochromated Mo-Kα radiation (λ = 0.71073 Å). Lorentz polarization, ω-φ scanning method and absorption corrections were utilized. The structures were analyzed by direct methods and refined with the full-matrix least-squares method utilizing the SHELXS-97 and SHELXL-97 programs. Anisotropic thermal parameters were assigned to all non-hydrogen atoms. Organic hydrogen atoms were defined geometrically. Analytical expressions of neutral-atom scattering factors were utilized and anomalous dispersion corrections were incorporated. Crystallographic data and refinement details for 1 and 2 were summarized in Table S1, selected bond lengths and angles were summarized in Table S2-S5 and selected hydrogen bonds lengths [Å] and angles [°] were calculated with the program PLATON and listed in Tables S6-S7. CCDC-2213806 (1), CCDC-2213805 (2) contain the crystallographic data for this work. These data can be obtained free of charge from Cambridge Crystallographic Data Centre via www.ccdc.cam.ac.uk/data_request/cif.

3. Experimental Section

Preparation of $[Eu_2(L)_3(H_2O)(DMF)]_n$ (1).

A mixture of Europium chloride (18.3 mg, 0.05 mmol) and H_2L (15.4 mg, 0.05 mmol) was added into the mixture of DMF (4 mL) and H_2O (3 mL). The mixed solutions were sealed in a 25 mL Teflon reactor, after the reaction mixture was heated at 120 °C for one day and then cooled to room temperature. After 48 hours reaction time, colorless bulky crystals of **1** were isolated in 65 % yield based on H_2L ligand. Elemental analysis found (%) for $C_{3.19}H_{2.25}Eu_{0.12}N_{0.62}O_{0.88}$: C 49.1 H 2.92 N 7.36; calcd: C 49.36, H 2.99, N 7.46.

Preparation of $[Tb_2(L)_3(H_2O)_2(DMF)]_n$ (2).

A mixture of Terbium chloride (74.6 mg, 0.2 mmol) and H_2L (31 mg, 0.1 mmol) was added into the mixture of DMF (2 mL) and H_2O (0.5 mL). The mixed solutions were sealed in a 25 mL Teflon reactor, after the reaction mixture was heated at 120 °C for one day and then cooled to room temperature. After 48 hours reaction time, colorless bulky crystals of **2** were isolated in 54 % yield based on H_2L ligand. Elemental analysis found (%) for $C_{48.75}H_{32.25}N_{9.25}O_{14}Tb_2$: C 47.98 H 2.96 N 7.18; calcd: C 48.19, H 3.07, N 7.28.

Preparation of $Eu_{0.096}Tb_{0.904}$-MOF (3).

A mixture of Europium chloride (2.7 mg, 40.1 nmol), Terbium chloride (53.1 mg, 0.2 mmol) and H_2L (46.2 mg, 0.15 mmol) was added into the mixture of DMF (1 mL) and H_2O (0.7 mL). The mixed solutions were sealed in a 25 mL Teflon reactor, after the reaction mixture was heated at 120 °C for one day and then cooled to room temperature naturally, colorless bulky crystals of **3** were isolated in 63 % yield based on H_2L ligand. The product was washed with ethanol to exchange residual DMF solvent molecules in the pores of Ln-MOF and then dried in a vacuum oven to volatilize all the solvent traces.

Preparation of $Eu_{0.051}Tb_{0.949}$-MOF (4).

A mixture of Europium chloride (3.66 mg, 0.01 mmol), Terbium chloride (70.87 mg, 0.19 mmol) and H_2L (31 mg, 0.1 mmol) was added into the mixture of DMF (2 mL) and H_2O (1 mL). The mixed solutions were sealed in a 25 mL Teflon reactor, after the reaction mixture was heated at 120 °C for one day and then cooled to room temperature naturally, colorless bulky crystals of **4** were isolated in 66 % yield based on H_2L ligand. The product was washed with ethanol to exchange residual DMF solvent molecules in the pores of Ln-MOF and then dried in a vacuum oven to volatilize all the solvent traces.

Preparation of $Eu_{0.011}Tb_{0.989}$-MOF (5).

A mixture of Europium chloride (0.732 mg, 0.002 mmol), Terbium chloride (13 mg, 0.19 mmol) and H_2L (31 mg, 0.1 mmol) was added into the mixture of DMF (2 mL) and H_2O (0.7 mL). The mixed solutions were sealed in a 25 mL Teflon reactor, after the reaction mixture was heated at 120 °C for one day and then cooled to room temperature naturally, colorless bulky crystals of **5** were isolated in 55 % yield based on H_2L ligand. The product was washed with ethanol to exchange residual DMF solvent molecules in the pores of Ln-MOF and then dried in a vacuum oven to volatilize all the solvent traces.

Preparation of $Eu_{0.415}Tb_{0.585}$-MOF (6).

A mixture of Europium chloride (36.6 mg, 0.1 mmol), Terbium chloride (37.3 mg, 0.1 mmol) and H_2L (31 mg, 0.1 mmol) was added into the mixture of DMF (2 mL) and H_2O (0.7 mL). The mixed solutions were sealed in a 25 mL Teflon reactor, after the reaction mixture was heated at 120 °C for one day and then cooled to room temperature naturally, colorless bulky crystals of **6** were isolated in 45 % yield based on H_2L ligand. The product was washed with ethanol to exchange residual DMF solvent molecules in the pores of Ln-MOF and then dried in a vacuum oven to

volatilize all the solvent traces.

Preparation of $Eu_{0.516}Tb_{0.484}$-MOF (7).

A mixture of Europium chloride (13 mg, 0.05 mmol), Terbium chloride (13 mg, 0.05 mmol) and H_2L (15.4 mg, 0.05 mmol) was added into the mixture of DMF (2 mL) and H_2O (0.7 mL). The mixed solutions were sealed in a 25 mL Teflon reactor, after the reaction mixture was heated at 120 °C for one day and then cooled to room temperature naturally, colorless bulky crystals of **7** were isolated in 55 % yield based on H_2L ligand. The product was washed with ethanol to exchange residual DMF solvent molecules in the pores of Ln-MOF and then dried in a vacuum oven to volatilize all the solvent traces.

4. Analysis of luminescent decay test of MOF 1-3@Hydroxyflutamidine after added AR

After adding different concentrations of AR, the luminescence lifetime of **MOF 1@Hydroxyflutamidine** (emission at 614 nm) decreased from 230 μs to 192 μs while the lifetime of **MOF 2@Hydroxyflutamidine** (emission at 543 nm) decreased from 209 μs to 186 μs. In addition, the lifetime of **MOF 3@Hydroxyflutamidine** at 614 nm decreased from 427 μs to 277 μs and **MOF 3@Hydroxyflutamidine** located at 543 nm increased from 72.9 μs to 100 μs. This confirms that AR can interrupt energy transfer between Tb^{3+} and Eu^{3+} in **MOF 3@Hydroxyflutamidine** (Table. S15).

Table S1. Crystallographic data and details of refinements for Eu-MOF (1) and Tb-MOF (2) [a,b]**.**

	Eu-MOF (1)	Tb-MOF (2)
Formula	$C_{3.19}H_{2.25}Eu_{0.12}N_{0.62}O_{0.88}$	$C_{48.75}H_{32.25}N_{9.25}O_{14}Tb_2$
M (g mol^{-1})	82.3	1289.43
Crystal system	Monoclinic	Monoclinic
Space group	C2/c	C 2/c
a (Å)	22.7369 (19)	22.681(5)
b (Å)	13.7847 (11)	13.694(3)
c (Å)	36.172 (3)	35.586(7)
α (deg)	90	90
β (deg)	100.8480(10))	101.482(4)
γ (deg)	90	90
V (Å^3)	11134.4(15)	10832(4)
Z	128	8
F (000)	5200	5052
ρ_{calc} (Mg m^{-3})	1.571	1.581
μ (mm^{-1})	2.303	2.660
data/restraints/parameters	11436 / 14 / 704	10884 / 70 / 698
GOF on F^2	1.099	1.005
R_1[a] (I = 2σ(I))	0.0474	0.1254

$\omega R_2{}^b$ (all data)	0.0984	0.3392

^{a} $R_1 = \Sigma||F_o| - |F_c||/|F_o|$. $^b\omega R_2 = [\Sigma w(|F_o|^2 - |F_c{}^2|^2/w|F_o{}^2|^2]^{1/2}$.

Table S2. Selected bond lengths [Å] for Eu-MOF (1).

Eu-MOF			
Eu1—O1	2.325 (4)	Eu2—O6^{i}	2.309 (4)
Eu1—O5	2.404 (4)	Eu2—O2	2.328 (4)
Eu1—O13	2.405 (5)	Eu2—O7^{iii}	2.388 (5)
Eu1—O14	2.418 (5)	Eu2—O11^{iv}	2.388 (4)
Eu1—O10	2.479 (4)	Eu2—O9	2.432 (4)
Eu1—O4^{i}	2.548 (4)	Eu2—O8^{iii}	2.432 (4)
Eu1—O3^{i}	2.556 (4)	Eu2—O3^{i}	2.450 (4)
Eu1—N9^{ii}	2.577 (5)	Eu2—O12^{iv}	2.516 (4)
Eu1—O9	2.608 (4)	O6—Eu2^{v}	2.310 (4)
O3—Eu2^{v}	2.450 (4)	O7—Eu2^{vi}	2.388 (5)
O3—Eu1^{v}	2.556 (4)	O8—Eu2^{vi}	2.432 (4)
O11—Eu2^{iv}	2.388 (4)	O12—Eu2^{iv}	2.516 (4)
N9—Eu1^{ii}	2.577 (5)		

Symmetry codes: (i) −x+3/2, y+1/2, −z+3/2; (ii) −x+2, −y+2, −z+2; (iii) x, y+1, z; (iv) −x+2, y, −z+3/2; (v) −x+3/2, y−1/2, −z+3/2; (vi) x, y−1, z.

Table S3. Selected angles [°] for Eu-MOF (1)

Eu-MOF			
O14—Eu1—O9	70.10 (15)	O1—Eu1—O10	127.02 (14)
O10—Eu1—O9	51.08 (12)	O5—Eu1—O10	128.87 (16)
O4^{i}—Eu1—O9	93.85 (13)	O13—Eu1—O10	139.93 (18)
O3^{i}—Eu1—O9	64.86 (13)	O14—Eu1—O10	72.44 (18)
N9^{ii}—Eu1—O9	123.25 (15)	O1—Eu1—O4^{i}	128.82 (15)
O4^{i}—Eu1—N9^{ii}	72.60 (15)	O1—Eu1—O5	81.80 (15)
O3^{i}—Eu1—N9^{ii}	123.36 (15)	O1—Eu1—O13	85.1 (2)
O1—Eu1—O9	77.11 (14)	O5—Eu1—O13	72.40 (19)
O5—Eu1—O9	139.20 (15)	O1—Eu1—O14	81.04 (18)
O13—Eu1—O9	138.5 (2)	O5—Eu1—O14	72.40 (17)
O1—Eu1—N9^{ii}	152.26 (17)	O13—Eu1—O14	143.60 (19)
O5—Eu1—N9^{ii}	70.54 (15)	O6^{i}—Eu2—O7^{iii}	87.94 (17)
O13—Eu1—N9^{ii}	88.4 (2)	O2—Eu2—O7^{iii}	156.46 (15)
O14—Eu1—N9^{ii}	88.52 (18)	O6^{i}—Eu2—O11^{iv}	126.66 (14)
O10—Eu1—N9^{ii}	72.63 (15)	O2—Eu2—O11^{iv}	79.65 (17)
O5—Eu1—O3^{i}	144.65 (14)	O7^{iii}—Eu2—O11^{iv}	89.28 (19)
O13—Eu1—O3^{i}	75.60 (18)	O6^{i}—Eu2—O9	141.57 (14)
O14—Eu1—O3^{i}	134.10 (15)	O2—Eu2—O9	77.26 (14)
O10—Eu1—O3^{i}	85.81 (15)	O7^{iii}—Eu2—O9	121.77 (15)
O4^{i}—Eu1—O3^{i}	50.85 (12)	O11^{iv}—Eu2—O9	81.18 (14)
O5—Eu1—O4^{i}	126.28 (15)	O6^{i}—Eu2—O8^{iii}	121.49 (15)
O13—Eu1—O4^{i}	68.84 (17)	O2—Eu2—O8^{iii}	148.96 (15)
O14—Eu1—O4^{i}	143.20 (17)	O7^{iii}—Eu2—O8^{iii}	52.70 (15)
O10—Eu1—O4^{i}	71.86 (15)	O11^{iv}—Eu2—O8^{iii}	97.56 (16)
O1—Eu1—O3^{i}	80.93 (15)	O9—Eu2—O8^{iii}	71.78 (14)
O6^{i}—Eu2—O12^{iv}	74.25 (14)	O6^{i}—Eu2—O3^{i}	78.03 (14)

O2—Eu2—O12^{iv}	78.32 (15)	O2—Eu2—O3^{i}	88.00 (15)
O7^{iii}—Eu2—O12^{iv}	78.42 (15)	O7^{iii}—Eu2—O3^{i}	110.93 (16)
O11^{iv}—Eu2—O12^{iv}	53.10 (13)	O11^{iv}—Eu2—O3^{i}	149.74 (14)
O9—Eu2—O12^{iv}	131.15 (13)	O9—Eu2—O3^{i}	69.13 (13)
O8^{iii}—Eu2—O12^{iv}	124.71 (16)	O8^{iii}—Eu2—O3^{i}	79.05 (15)
O3^{i}—Eu2—O12^{iv}	150.41 (14)		

Symmetry codes: (i) −x+3/2, y+1/2, −z+3/2; (ii) −x+2, −y+2, −z+2; (iii) x, y+1, z; (iv) −x+2, y, −z+3/2; (v) −x+3/2, y−1/2, −z+3/2; (vi) x, y−1, z.

Table S4. Selected bond lengths [Å] for Tb-MOF (2).

**Tb-MOF**			
Tb1—O11^{i}	2.283 (14)	Tb2—O2^{iii}	2.306 (13)
Tb1—O1	2.326 (13)	Tb2—O12^{iv}	2.317 (14)
Tb1—O6	2.340 (13)	Tb2—O13	2.386 (16)
Tb1—O3^{ii}	2.381 (13)	Tb2—O14'	2.409 (10)
Tb1—O10	2.398 (16)	Tb2—O14	2.411 (10)
Tb1—O9	2.439 (14)	Tb2—O4^{v}	2.506 (12)
Tb1—O8^{iii}	2.445 (12)	Tb2—O7	2.508 (15)
Tb1—O5	2.500 (13)	Tb2—O8	2.534 (12)
Tb2—N9^{vi}	2.549 (13)	Tb2—O3^{v}	2.549 (12)

Symmetry codes: (i) −*x*+1/2, *y*−1/2, −*z*+1/2; (ii) −*x*+1/2, *y*+1/2, −*z*+1/2; (iii) −*x*, *y*, −*z*+1/2; (iv) −*x*, *y*−1, −*z*+1/2; (v) *x*−1/2, *y*+1/2, *z*; (vi) *x*−1/2, −*y*+3/2, *z*−1/2; (vii) *x*+1/2, *y*−1/2, *z*; (viii) −*x*, *y*+1, −*z*+1/2; (ix) *x*+1/2, −*y*+3/2, *z*+1/2.

Table S5. Selected angles [º] for Tb-MOF (1)

Tb-MOF			
O11^{i}—Tb1—O1	83.5 (5)	O9—Tb1—O8^{iii}	71.7 (4)
O11^{i}—Tb1—O6	127.8 (5)	O11^{i}—Tb1—O5	73.6 (5)
O1—Tb1—O6	79.0 (5)	O1—Tb1—O5	77.5 (5)
O11^{i}—Tb1—O3^{ii}	75.8 (5)	O6—Tb1—O5	54.8 (5)
O1—Tb1—O3^{ii}	91.4 (5)	O3^{ii}—Tb1—O5	148.4 (5)
O6—Tb1—O3^{ii}	152.3 (5)	O10—Tb1—O5	77.8 (5)
O11^{i}—Tb1—O10	85.6 (6)	O9—Tb1—O5	124.3 (5)
O1—Tb1—O10	155.0 (5)	O8^{iii}—Tb1—O5	134.7 (4)
O6—Tb1—O10	90.2 (6)	O2^{iii}—Tb2—O12^{iv}	81.5 (5)
O3^{ii}—Tb1—O10	107.6 (5)	O2^{iii}—Tb2—O13	85.9 (6)
O11^{i}—Tb1—O9	121.0 (5)	O12^{iv}—Tb2—O13	72.6 (6)
O1—Tb1—O9	149.5 (5)	O2^{iii}—Tb2—O14'	84.0 (7)
O6—Tb1—O9	96.3 (6)	O12^{iv}—Tb2—O14'	73.1 (8)
O3^{ii}—Tb1—O9	78.9 (5)	O13—Tb2—O14'	145.3 (8)
O10—Tb1—O9	53.4 (5)	O2^{iii}—Tb2—O14	75.4 (8)
O11^{i}—Tb1—O8^{iii}	139.3 (4)	O12^{iv}—Tb2—O14	67.6 (19)
O1—Tb1—O8^{iii}	77.8 (5)	O13—Tb2—O14	137.9 (18)
O6—Tb1—O8^{iii}	83.6 (4)	O2^{iii}—Tb2—O4^{v}	129.7 (5)
O3^{ii}—Tb1—O8^{iii}	69.0 (4)	O12^{iv}—Tb2—O4^{v}	127.8 (5)
O10—Tb1—O8^{iii}	123.8 (5)	O13—Tb2—O4^{v}	70.2 (6)
O2^{iii}—Tb2—O8	76.6 (4)	O14'—Tb2—O4^{v}	138.6 (7)
O12^{iv}—Tb2—O8	139.3 (5)	O14—Tb2—O4^{v}	148.4 (13)
O13—Tb2—O8	138.1 (5)	O2^{iii}—Tb2—O7	126.9 (5)
O14'—Tb2—O8	70.8 (7)	O12^{iv}—Tb2—O7	128.7 (5)
O14—Tb2—O8	73.8 (18)	O13—Tb2—O7	139.6 (6)
O4^{v}—Tb2—O8	92.2 (4)	O14'—Tb2—O7	69.7 (7)

O7—Tb2—O8	51.6 (4)	O14—Tb2—O7	79.0 (16)
O2^{iii}—Tb2—O3^{v}	80.4 (5)	O4^{v}—Tb2—O7	70.3 (5)
O12^{iv}—Tb2—O3^{v}	143.5 (5)	O14'—Tb2—N9^{vi}	87.5 (7)
O13—Tb2—O3^{v}	74.7 (5)	O14—Tb2—N9^{vi}	93.0 (10)
O14'—Tb2—O3^{v}	135.4 (7)	O4^{v}—Tb2—N9^{vi}	72.4 (4)
O14—Tb2—O3^{v}	136.0 (15)	O7—Tb2—N9^{vi}	75.1 (5)
O4^{v}—Tb2—O3^{v}	51.3 (4)	O8—Tb2—N9^{vi}	126.4 (4)
O7—Tb2—O3^{v}	87.2 (5)	O3^{v}—Tb2—N9^{vi}	123.7 (4)
O8—Tb2—O3^{v}	65.1 (4)	O12^{iv}—Tb2—N9^{vi}	69.2 (4)
O2^{iii}—Tb2—N9^{vi}	150.8 (5)	O13—Tb2—N9^{vi}	85.4 (6)

Symmetry codes: (i) −*x*+1/2, *y*−1/2, −*z*+1/2; (ii) −*x*+1/2, *y*+1/2, −*z*+1/2; (iii) −*x*, *y*, −*z*+1/2; (iv) −*x*, *y*−1, −*z*+1/2; (v) *x*−1/2, *y*+1/2, *z*; (vi) *x*−1/2, −*y*+3/2, *z*−1/2; (vii) *x*+1/2, *y*−1/2, *z*; (viii) −*x*, *y*+1, −*z*+1/2; (ix) *x*+1/2, −*y*+3/2, *z*+1/2.

Table S6. Hydrogen bonds for Eu-MOF [Å and °][a]

D-H···A	d(D-H)	d(H···A)	d(D···A)	<(DHA)
O(13)-H(13A)···O(4)	0.96	2.33	2.803(7)	110
O(13)-H(13B)···O(7)	0.96	2.09	3.032(8)	164
C(7)-H(7)···O(6)	0.93	2.31	3.224(7)	170
C(16)-H(16)···O(4)	0.93	2.47	3.377(9)	165
C(29)-H(29)···N(5)	0.93	2.50	2.810(10)	100
C(32)-H(32)···O(14)	0.93	2.56	3.391(14)	148
C(35)-H(35)···N(3)	0.93	2.60	3.482(12)	159
C(39)-H(39)···O(11)	0.93	2.42	3.224(7)	145
C(45)-H(45)···N(8)	0.93	2.53	2.837(10)	100
C(48)-H(48)···O(10)	0.93	2.40	2.944(8)	117

Table S7. Hydrogen bonds for Tb-MOF [Å and °][a]

D-H···A	d(D-H)	d(H···A)	d(D···A)	<(DHA)
O(13)-H(13A)···O(12)	0.97	2.41	2.79(2)	102
O(13)-H(13A)···O(5)	0.97	1.91	2.80(2)	151
O(13)-H(13B)···O(4)	0.96	2.39	2.81(2)	106
O(14')-H(14A)···O(12)	0.97	2.46	2.82(3)	101
O(14')-H(14B)···O(8)	0.96	2.49	2.87(3)	103
C(5)-H(5)···O(4)	0.93	2.53	2.83(2)	100
C(7)-H(7)···O(11)	0.93	2.28	3.19(2)	165
C(19)-H(19)···O(8)	0.93	2.43	2.79(2)	103
C(37)-H(37)···O(12)	0.93	2.46	2.78(2)	100
C(48)-H(48)···O(7)	0.93	2.56	3.08(3)	116

Table. S8. Limit of detection (LOD) value for the detection of Hydroxyflutamine in different work.

Sensor name	Detection method	LOD	Ref
pespsin-CuNCs	Fluorescence	61.82 nM	1
CB/β-CD	Fluorescence	0.016 μM	2
Re–Ru@f-MWCNT/GCE	Electrochemical method	3.17 nM	3
MoS_2-CZO/GCE	Electrochemical method	0.005 μM	4
nano - Ag/MGCE	Electrochemical method	9.33 μM	5
BDDE	Electrochemical method	0.42 μM	6
Sn-ZnO-rGO	Electrochemical method	7.3 nM	7
Eu/Tb-MOF	Fluorescence	8.37 fM	This work

Table S9. Results of Hydroxyflutamide detection based on MOF 3 in human serum solution (10%, water as solvent) (n=3)

Sample	Added(pM)	Found(pM)	Recovery(%)	RSD(%)
1	0	Not detected		
2	0.02	0.0194	97	0.056
3	0.04	0.0382	95.3	0.053
4	0.06	0.0588	98	0.048
5	0.08	0.0764	95.5	0.06
6	0.1	0.1035	103.5	0.05
7	0.12	0.1267	105.6	0.0656
8	0.14	0.1473	105.2	0.0553
9	0.2	0.2108	105.4	0.053
10	0.22	0.2174	98.8	0.048
11	0.24	0.2337	97.4	0.06
12	0.26	0.2517	96.8	0.05

Table S10 Amplitude averaged luminescent lifetime of MOF 1-3 after adding different concentration of hydroxyflutamide, excitation position is 266 nm.

Sample Name	Wavelength /nm	Lifetime /μs
MOF 3	543	714.13
MOF 3 + Hydroxyflutamide (480.9 ng/mL)	543	664.22
MOF 3	614	427.69
MOF 3 + Hydroxyflutamide (480.9 ng/mL)	614	352.72
MOF 1	614	131
MOF 1+ Hydroxyflutamide	614	129.2
MOF 2	543	225.31
MOF 2+ Hydroxyflutamide	543	212.80

Table S11 Results of PSA detection based on MOF 3@Hydroxyflutamidine in human serum (n=3)

Sample	Added(pM)	Found(pM)	Recovery(%)	RSD(%)
1	0	Not detected		
2	2	1.99	99.5	0.825
3	4	4.11	102.75	0.975
4	6	5.87	97.8	0.865
5	8	8.03	100.38	0.651
6	10	9.38	93.8	0.778
7	12	11.89	99.08	0.6
8	14	14.13	100.93	0.601
9	16	16.05	100.31	0.66
10	18	18.16	100.89	0.778
11	20	21.08	105.4	0.822
12	22	21.74	98.8	0.847
13	24	23.37	97.4	0.825
14	26	25.17	96.8	0.691
15	28	27.21	97.18	0.8
16	30	30.12	100.4	0.975

Table S12. Results of AR detection based on MOF 3@Hydroxyflutamidine in human serum (n=3)

Sample	Added(pM)	Found(pM)	Recovery(%)	RSD(%)
1	0	Not detected		
2	1	1.048	104.8	1.965
3	3	2.931	97.69	2.438
4	4	3.93	98.25	2.298
5	5	5.003	100.06	2.642
6	6	5.898	98.3	3.108
7	7	6.792	97.03	3.012
8	8	7.949	99.4	2.7654
9	9	8.892	98.9	2.3497
10	10	10.092	100.92	2.4321
11	11	10.613	96.5	2.8453
12	12	11.713	97.6	2.8768
13	13	13.979	107.5	2.5689

Table. S13. Limit of detection (LOD) value for the detection of PSA in previous work.

Sample	Detection method	LOD	Ref
GR–Au	Electrochemical detection	0.59 ng/mL	8
TCNQ-$Cu_3(BTC)_2$ SPEs	Electrochemical detection	0.06 ng/mL	9
Silica nanoparticles	Electrochemical detection	0.76 ng/mL	10
Cd-MOF	Fluorescence sensor	27 pg/mL	11
QD-655	Fluorescence sensor	100 pg/mL	12
Eu/Tb-MOF	Fluorescence sensor	15.9 pg/mL	This work

Table. S14 Amplitude averaged luminescent lifetime of MOF 1-3@hydroxyflutamide after adding different concentration of PSA, excitation position is 266 nm.

Sample name	Wavelength/nm	LIfetime/μs
Hydroxyflutamide@MOF3	543	72.96
Hydroxyflutamide@MOF3 + PSA(10 ng/mL)	543	107.36
Hydroxyflutamide@MOF3 + PSA(20 ng/mL)	543	106.72
Hydroxyflutamide@MOF3 + PSA(30 ng/mL)	543	125.43
Hydroxyflutamide@MOF3	614	156.27
Hydroxyflutamide@MOF3+ PSA(30 ng/mL)	614	136.76
MOF1 @hydroxyflutamide	614	234.27
MOF1 @hydroxyflutamide+PSA(10 ng/mL)	614	219.27
Hydroxyflutamide@MOF1+PSA(20 ng/mL)	614	215.16
Hydroxyflutamide@MOF1+ PSA(30 ng/mL)	614	189.90
Hydroxyflutamide@MOF2	543	208.74
Hydroxyflutamide@MOF2+PSA(10 ng/mL)	543	203.00
Hydroxyflutamide@MOF2+PSA(20 ng/mL)	543	199.84
Hydroxyflutamide@MOF2+PSA(30 ng/mL)	543	194.34

Table. S15 Amplitude averaged luminescent lifetime of MOF 1-3@hydroxyflutamide after adding different concentration of AR, excitation position is 266 nm.

Sample Name	Wavelength /nm	Lifetime /μs
Hydroxyflutamide@MOF3	614	427.69
Hydroxyflutamide@MOF3+Ar (6 nM)	614	293.74
Hydroxyflutamide@MOF3+Ar (9 nM)	614	277.58
Hydroxyflutamide@MOF3	543	72.96
Hydroxyflutamide@MOF3+Ar (3 nM)	543	75.32
Hydroxyflutamide@MOF3+Ar (6 nM)	543	94.69
Hydroxyflutamide@MOF3+Ar (9 nM)	543	100.07
Hydroxyflutamide@MOF2	543	209.50
Hydroxyflutamide@MOF2+Ar (3 nM)	543	204.50
Hydroxyflutamide@MOF2+Ar (6 nM)	543	190.55
Hydroxyflutamide@MOF2+Ar (9 nM)	543	186.45
Hydroxyflutamide@MOF1	614	230.55
Hydroxyflutamide@MOF1+Ar (3 nM)	614	219.55
Hydroxyflutamide@MOF1+Ar (6 nM)	614	206.24
Hydroxyflutamide@MOF1+Ar (9 nM)	614	191.57

Table S16 Amplitude averaged luminescent lifetime of MOF 1-3 after adding different concentration of AR or PSA, excitation position is 266 nm.

Sample Name	Wavelength /nm	Lifetime /ns
Hydroxyflutamide@MOF3 (480.9 ng/mL)	350	1.51
Hydroxyflutamide@MOF3 (480.9 ng/mL) + AR (3 nM)	350	1.44
Hydroxyflutamide@MOF3 (480.9 ng/mL) + AR (6 nM)	350	1.36
Hydroxyflutamide@MOF3 (480.9 ng/mL)	350	1.67
Hydroxyflutamide@MOF3 (480.9 ng/mL) + PSA (50 ng/mL)	350	1.64

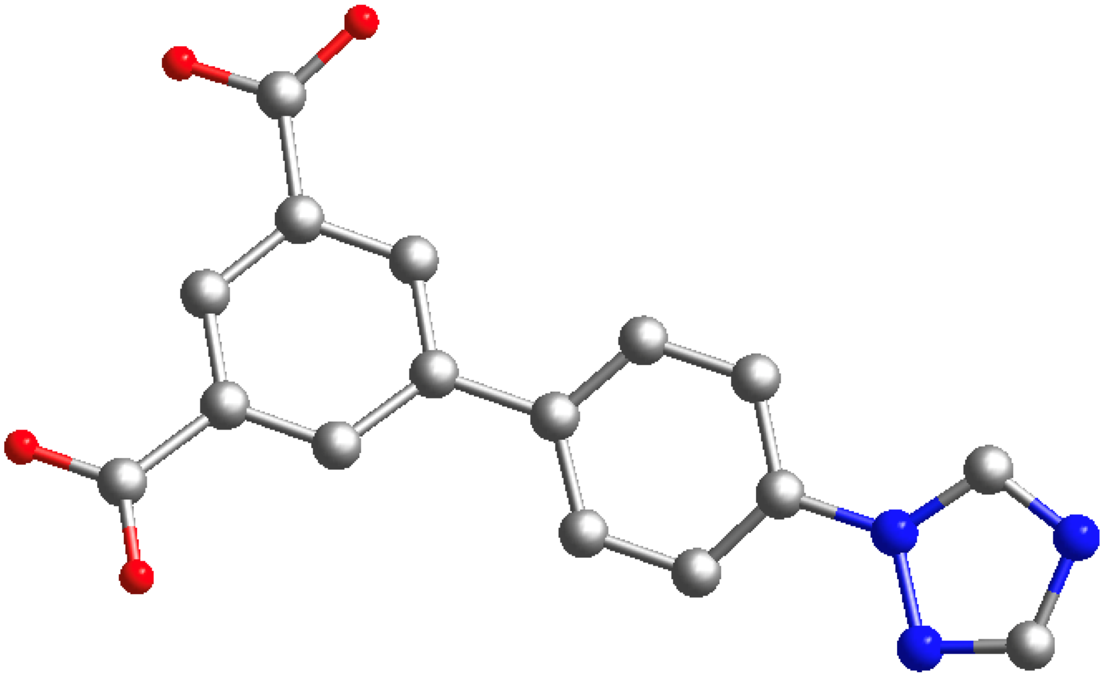

Figure. S1 Structure motif picture of H_2L (Carobon= Grey, Oxygen= Red, Nitrogen= Blue).

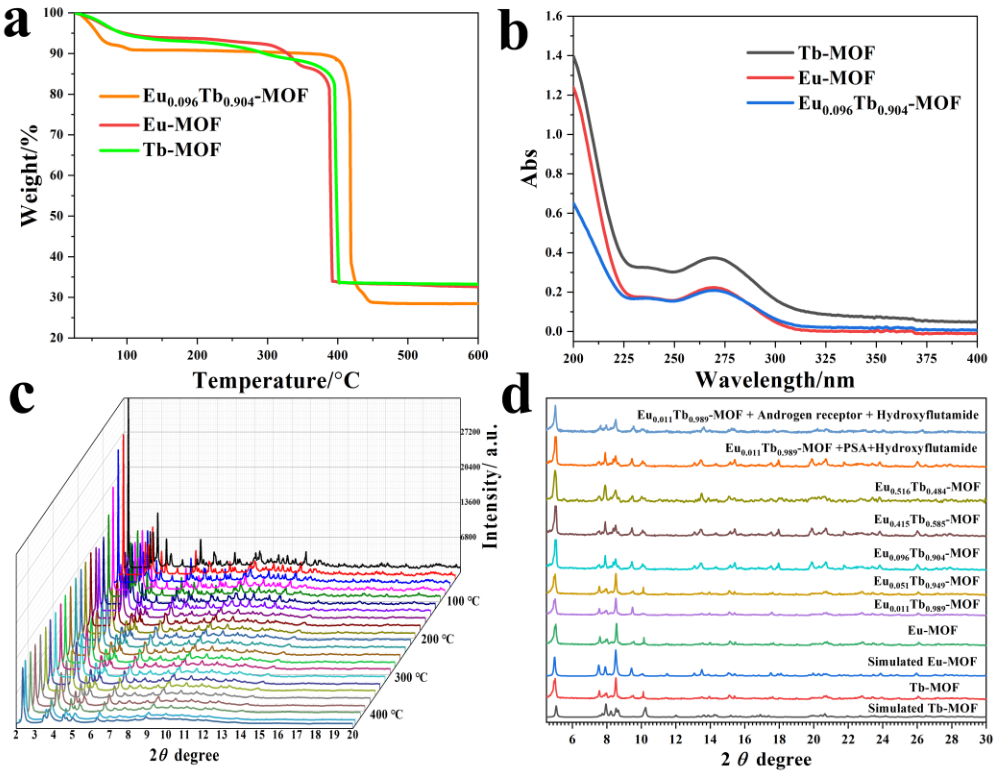

Figure. S2 (a) TG analysis of Eu-MOF, Tb-MOF and MOF 3. (b) UV-vis spectra of Eu-MOF, Tb-MOF and MOF 3. (c) Variable temperature Powder X-ray Diffraction (PXRD) patterns of MOF 3. (d) PXRD of Eu_x-Tb_{1-x}-MOF under different conditions.

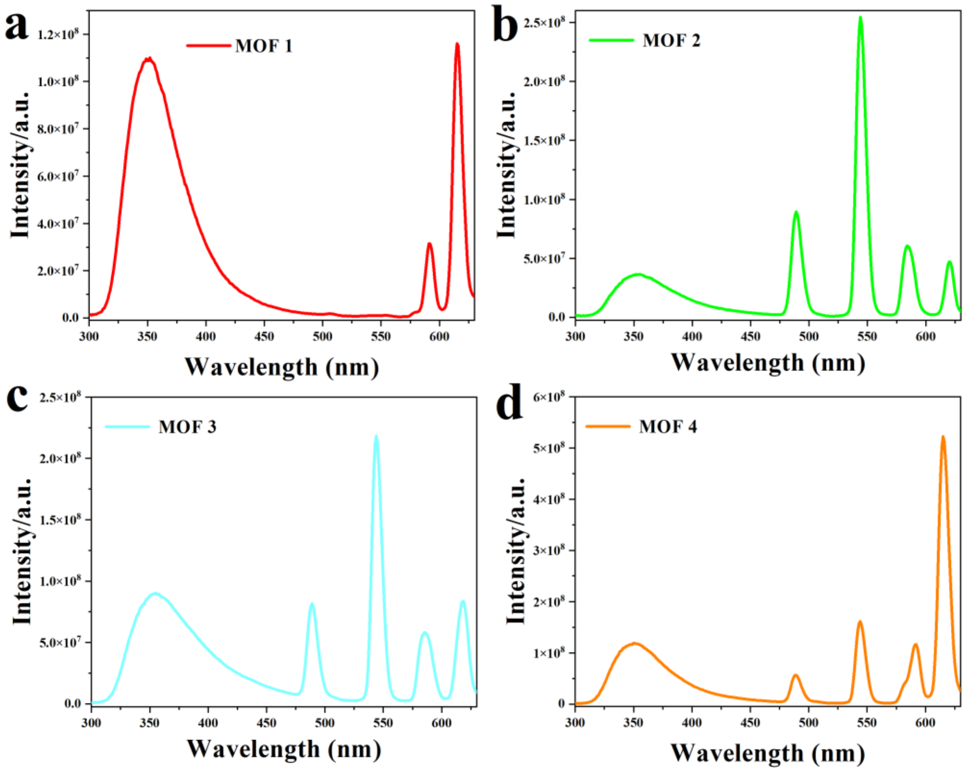

Figure. S3. Luminescence spectra of (a) MOF 1 suspension (0.1 g/L in water); (b) MOF 2 suspension (0.1 g/L in water); (c) MOF 3 suspension (0.1 g/L in water); (d) MOF 4 suspension (0.1 g/L in water).

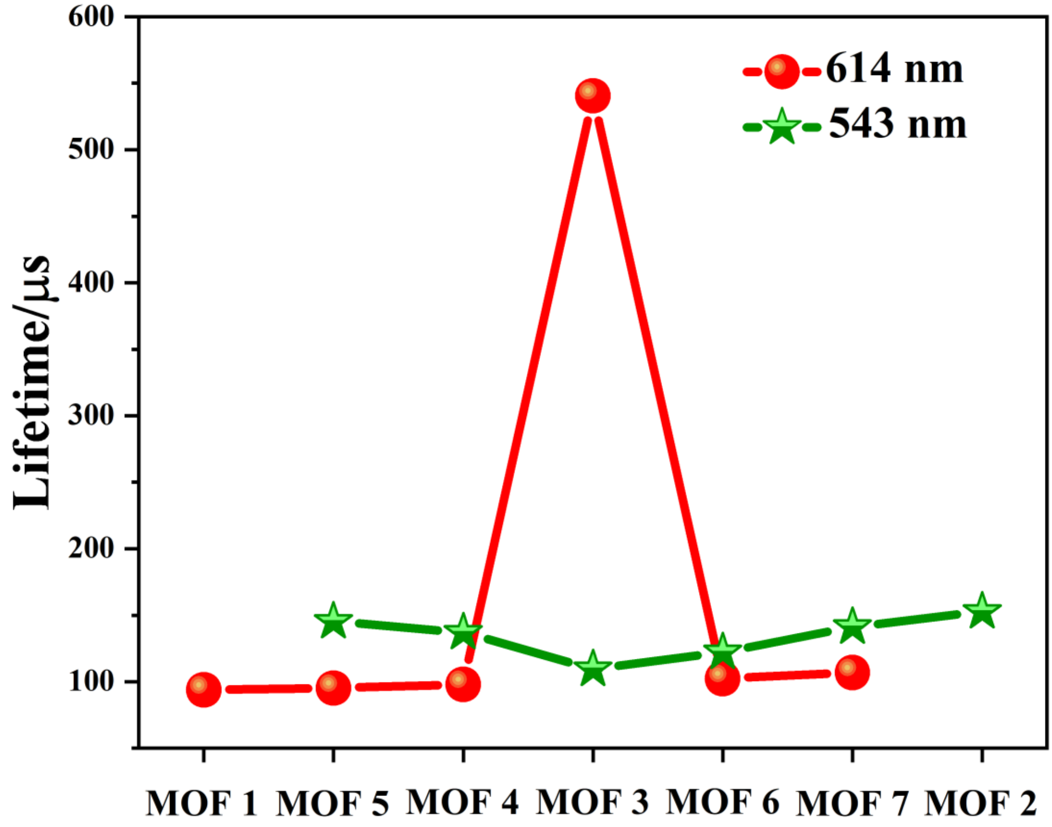

Figure. S4 Luminescence lifetime of MOF **1-7** at 543 nm and 614 nm (excitation at 260 nm).

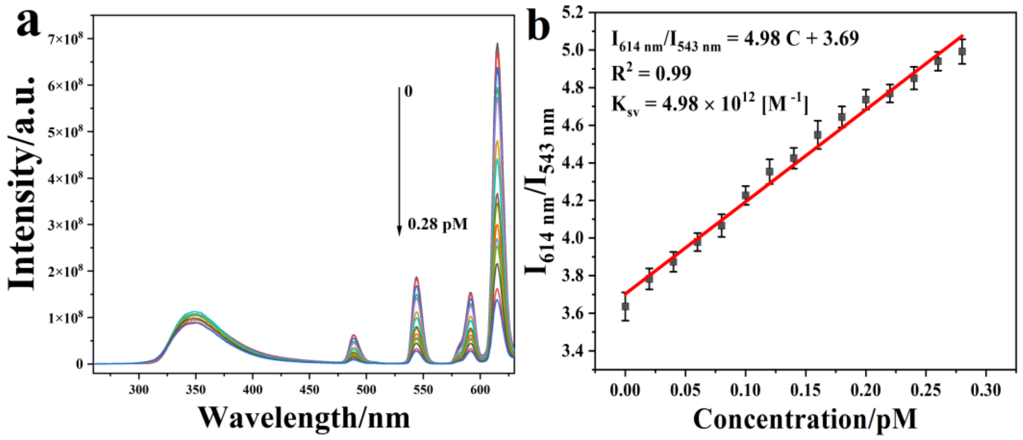

Figure. S5 (a) Luminescence spectra of **3** after adding Hydroxyflutamide at different concentrations (excitation: 250 nm); (b) linear relationship between concentration of Hydroxyflutamide and relative luminescence intensity ratio of **3**.

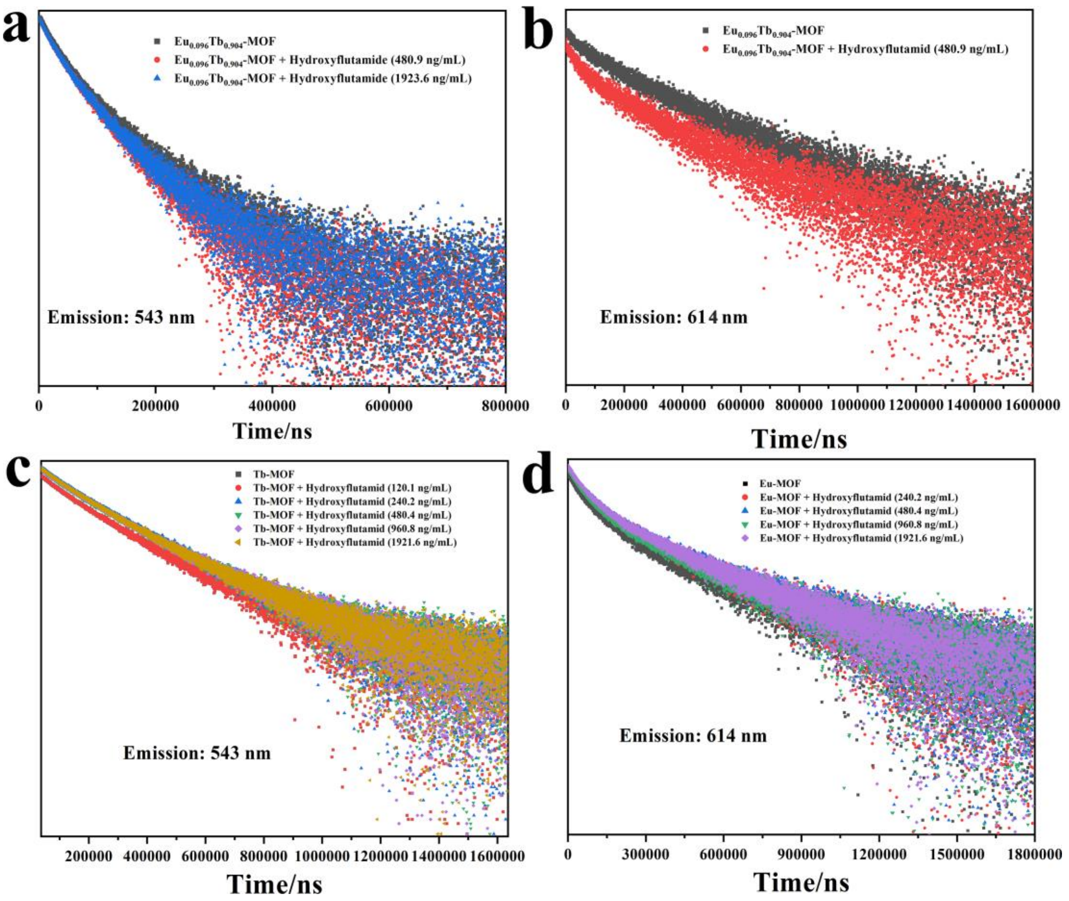

Figure. S6. The luminescence decays of (a) **MOF 1** (emission at 543 nm); (b) **MOF 2** (emission at 614 nm); (c) **MOF 3@hydroxyflutamide** (emission at 543 nm); (d) **MOF 3@hydroxyflutamide** (emission at 614 nm) after adding different concentrations of AR. All the experiments were performed at λ_{ex}=260 nm.

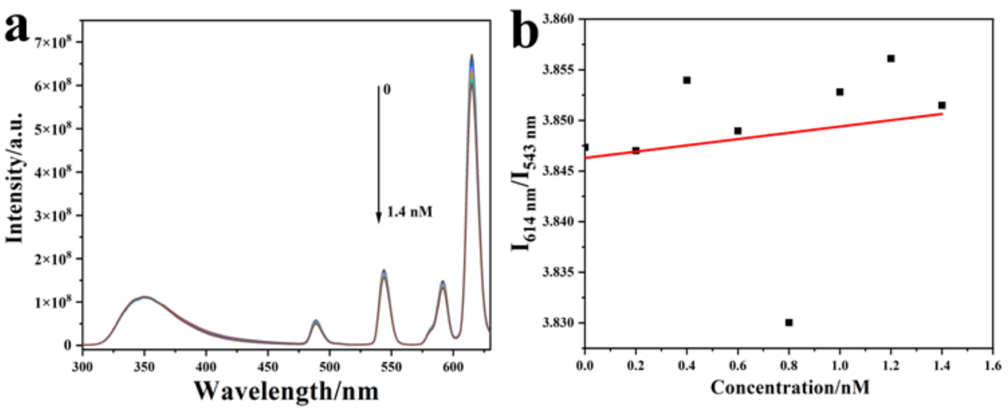

Figure. S7 Luminescence spectra of **3** after adding different concentrations of AR (excitation: 250 nm).

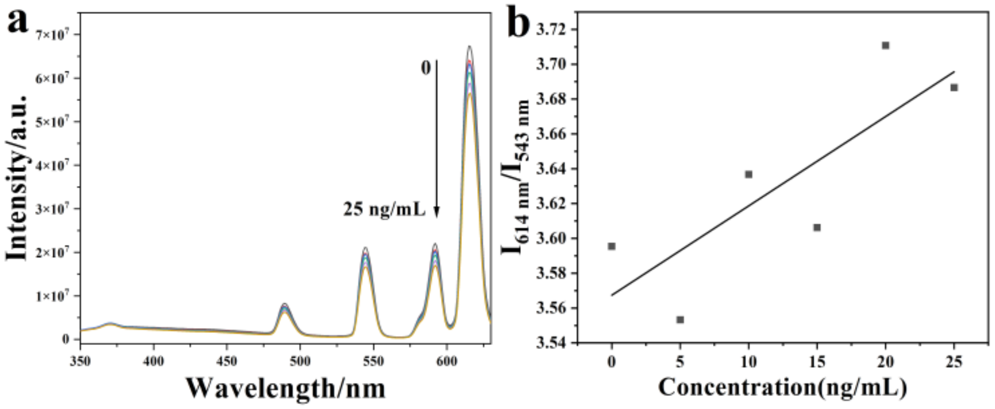

Figure. S8 Luminescence spectra of **3** after adding different concentrations of PSA (excitation: 250 nm).

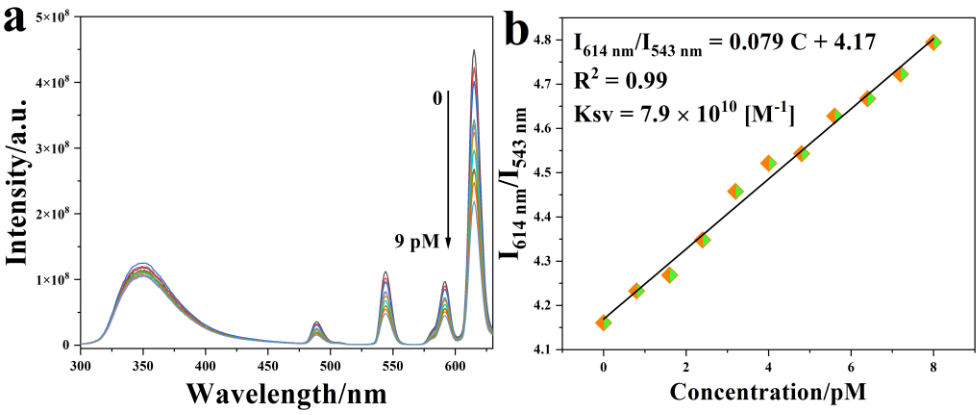

Figure. S9 (a) Luminescence spectra of **3** with Hydroxyflutamide (10 μM) after adding different concentrations of PSA (excitation: 250 nm); (b) the relationship between luminescent ratio of **3** and the concentration of PSA.

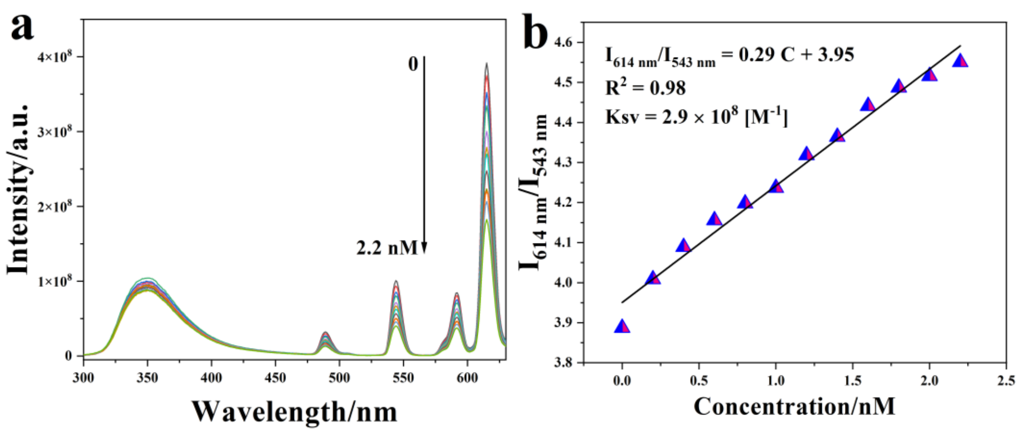

Figure. S10 (a) Luminescence spectra of **3** with (10 μM) after adding different concentrations of AR (excitation: 250 nm); (b) the relationship between luminescent ratio of **3** and the concentration of AR.

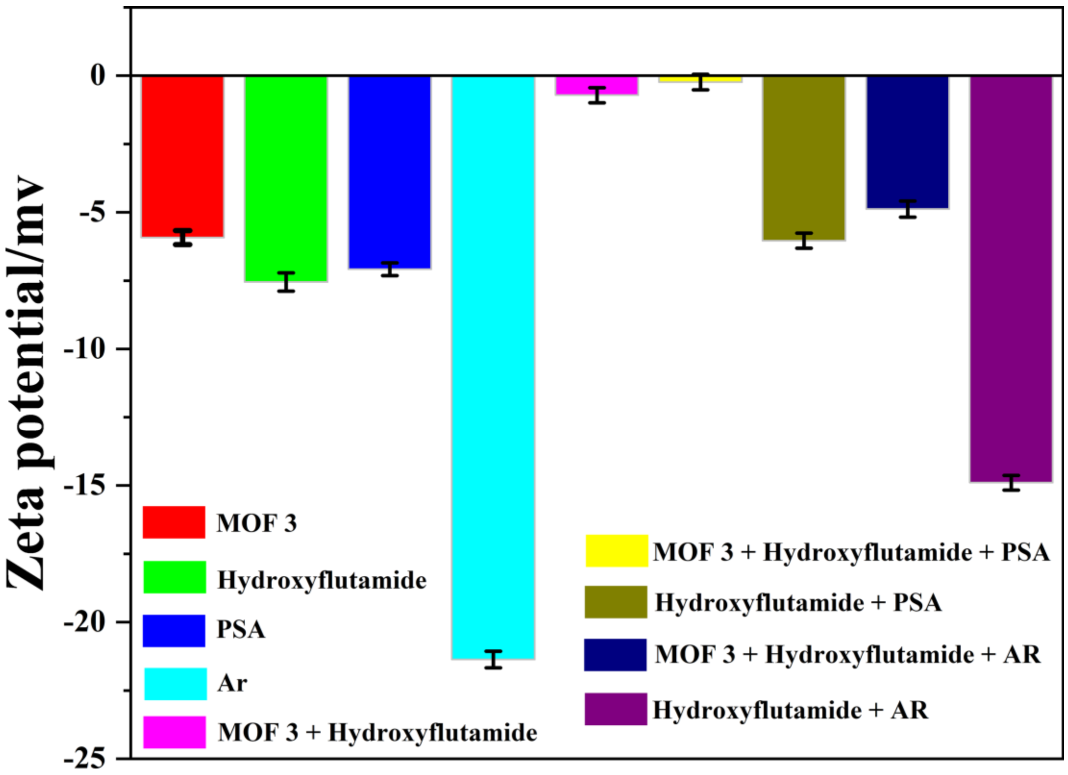

Figure. S11 Zeta potentials of MOF **3**, hydroflutamide, and the mixture of the MOF **3** with hydroxyflutamide and specific antigen and androgen receptor.

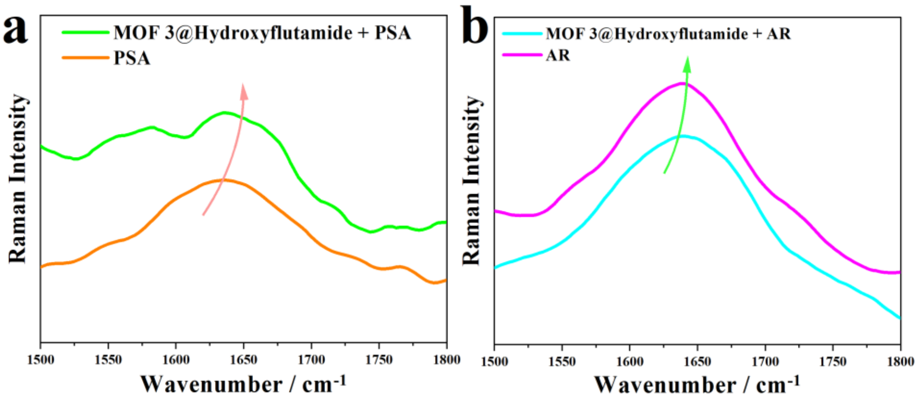

Figure. S12 Raman spectra of (a) PSA under different condition; (b) AR under different condition.

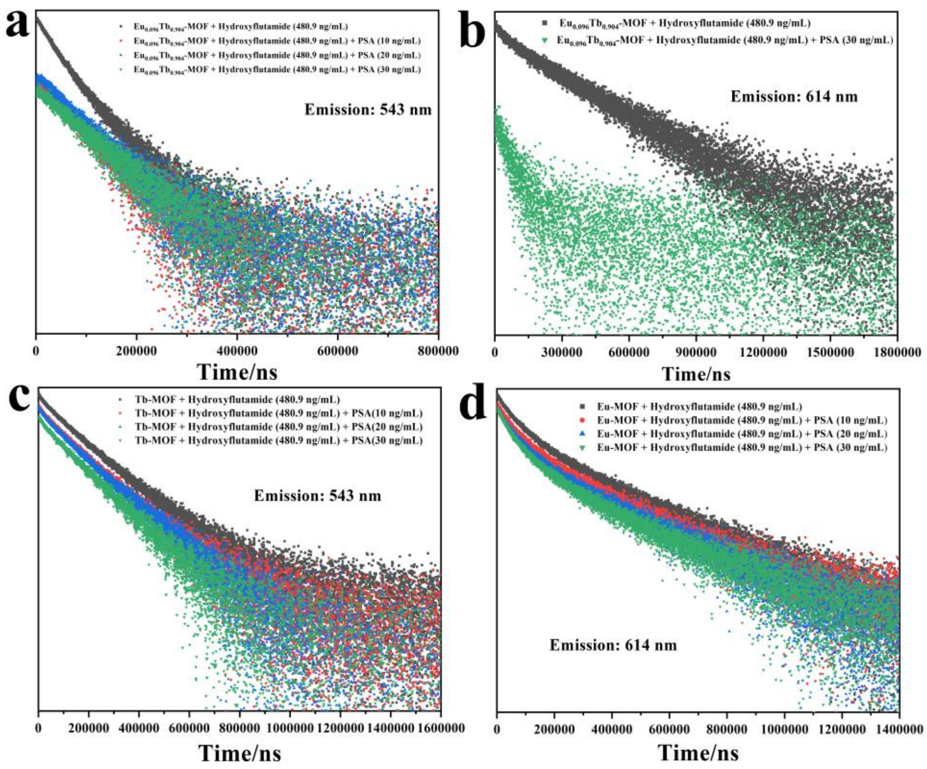

Figure. S13. The luminescence decays of (a) **MOF 3@Hydroxyflutamide** (emission at 543 nm); (b) **MOF3@Hydroxyflutamide** (emission at 614 nm); (c) **MOF2@Hydroxyflutamide (**emission at 543 nm); (d) **MOF1@Hydroxyflutamide** (emission at 614 nm) after adding different concentrations of PSA. All the experiments were performed at λ_{ex}=260 nm.

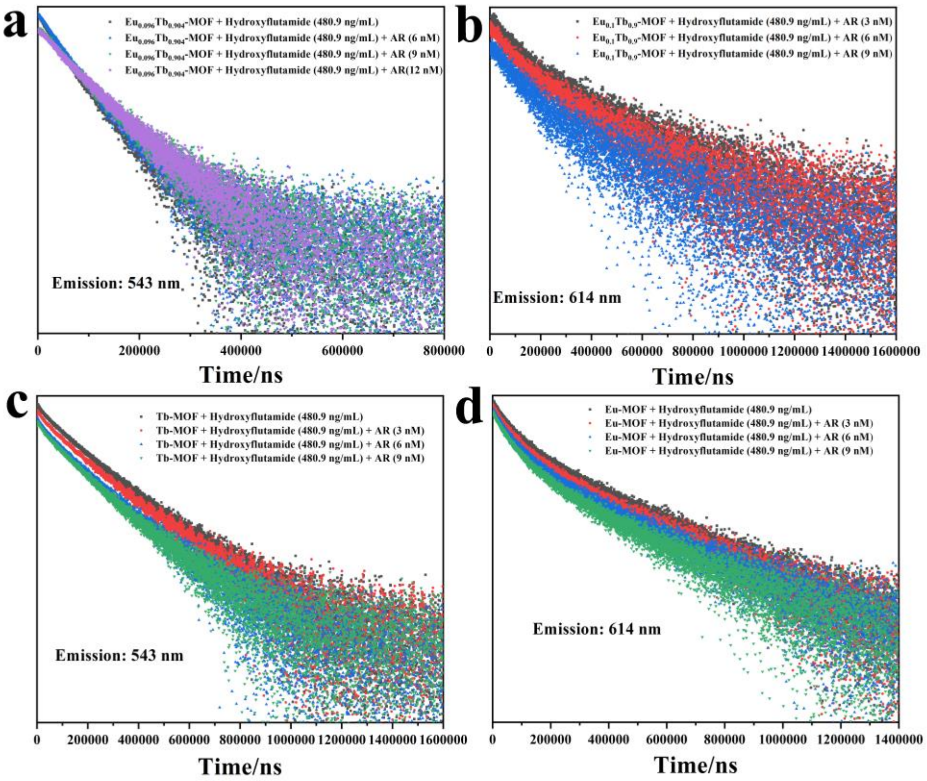

Figure. S14. The luminescence decays of (a) **MOF 3@Hydroxyflutamide** (emission at 543 nm); (b) **MOF 3@Hydroxyflutamide** (emission at 614 nm); (c) **MOF 2@ Hydroxyflutamide** (emission at 543 nm); (d) **MOF 1@Hydroxyflutamide** (emission at 614 nm) after adding different concentrations of AR. All the experiments were performed at λ_{ex}=260 nm.

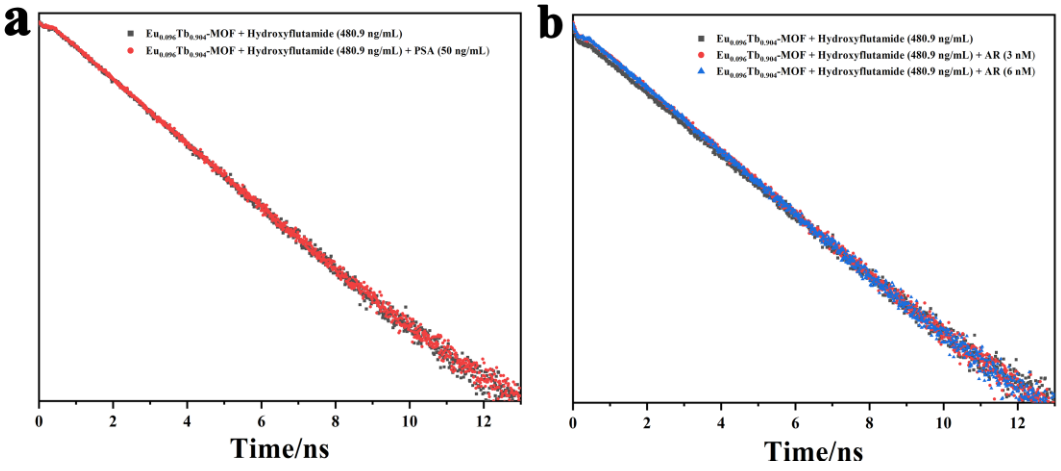

Figure. S15. The luminescence decays of (a) **MOF 3@Hydroxyflutamide** (emission at 350 nm) after adding different concentrations of PSA; and (b) **MOF 3@Hydroxyflutamide** (emission at 350 nm) after adding different concentrations of AR. All the experiments were performed at λ_{ex}= 260 nm.

References

1. S. Borse, Z. V. P. Murthy, T.-J. Park and S. K. Kailasa, *Microchemical Journal*, 2021, **164**.
2. S. Kubendhiran, R. Sakthivel, S. M. Chen, B. Mutharani and T. W. Chen, *Anal Chem*, 2018, **90**, 6283-6291.
3. P. Veerakumar, V. Vinothkumar, S.-M. Chen, A. Sangili and K.-C. Lin, *Journal of Materials Chemistry C*, 2021, **9**, 15949-15966.
4. S. V. Selvi, N. Nataraj, T. W. Chen, S. M. Chen, S. Nagarajan, C. S. Ko, T. W. Tseng and C. C. Huang, *Materials Today Chemistry*, 2022, **23**.
5. F. Ahmadi, J. B. Raoof, R. Ojani, M. Baghayeri, M. M. Lakouraj and H. Tashakkorian, *Chinese Journal of Catalysis*, 2015, **36**, 439-445.
6. Ľ. Švorc, K. Borovská, K. Cinková, D. M. Stanković and A. Planková, *Electrochimica Acta*, 2017, **251**, 621-630.
7. K.-Y. Hwa, A. Santhan and S. K. S. Tata, *Microchemical Journal*, 2021, **160**.
8. H. D. Jang, S. K. Kim, H. Chang and J. W. Choi, *Biosens Bioelectron*, 2015, **63**, 546-551.
9. S. K. Bhardwaj, A. L. Sharma, N. Bhardwaj, M. Kukkar, A. A. S. Gill, K.-H. Kim and A. Deep, *Sensors and Actuators B: Chemical*, 2017, **240**, 10-17.
10. B. Qu, X. Chu, G. Shen and R. Yu, *Talanta*, 2008, **76**, 785-790.
11. M. M. Wanderley, C. Wang, C. D. Wu and W. Lin, *J Am Chem Soc*, 2012, **134**, 9050-9053.
12. H. Y. Song, T. I. Wong, A. Sadovoy, L. Wu, P. Bai, J. Deng, S. Guo, Y. Wang, W. Knoll and X. Zhou, *Lab Chip*, 2015, **15**, 253-263.
